# Impact of an Electronic Health Record–Based Interruptive Alert Among Patients With Headaches Seen in Primary Care: Cluster Randomized Controlled Trial

**DOI:** 10.2196/58456

**Published:** 2024-08-29

**Authors:** Apoorva Pradhan, Eric A Wright, Vanessa A Hayduk, Juliana Berhane, Mallory Sponenberg, Leeann Webster, Hannah Anderson, Siyeon Park, Jove Graham, Scott Friedenberg

**Affiliations:** 1 Center for Pharmacy Innovation and Outcomes, Geisinger, Danville, PA, United States; 2 Department of Bioethics and Decision Sciences, Geisinger, Danville, PA, United States; 3 Pharmacy Support Services, Geisinger, Danville, PA, United States; 4 Health Information Technology, Geisinger, Danville, PA, United States; 5 Enterprise Pharmacy, Geisinger, Danville, PA, United States; 6 Pharmesol Inc, Auburndale, MA, United States; 7 Department of Neurology, Neuroscience Institute, Geisinger and Geisinger Commonwealth School of Medicine, Danville, PA, United States

**Keywords:** headache management, migraine management, electronic health record–based alerts, primary care, clinician decision support tools, electronic health record, EHR

## Abstract

**Background:**

Headaches, including migraines, are one of the most common causes of disability and account for nearly 20%‐30% of referrals from primary care to neurology. In primary care, electronic health record–based alerts offer a mechanism to influence health care provider behaviors, manage neurology referrals, and optimize headache care.

**Objective:**

This project aimed to evaluate the impact of an electronic alert implemented in primary care on patients’ overall headache management.

**Methods:**

We conducted a stratified cluster-randomized study across 38 primary care clinic sites between December 2021 to December 2022 at a large integrated health care delivery system in the United States. Clinics were stratified into 6 blocks based on region and patient-to–health care provider ratios and then 1:1 randomized within each block into either the control or intervention. Health care providers practicing at intervention clinics received an interruptive alert in the electronic health record. The primary end point was a change in headache burden, measured using the Headache Impact Test 6 scale, from baseline to 6 months. Secondary outcomes included changes in headache frequency and intensity, access to care, and resource use. We analyzed the difference-in-differences between the arms at follow-up at the individual patient level.

**Results:**

We enrolled 203 adult patients with a confirmed headache diagnosis. At baseline, the average Headache Impact Test 6 scores in each arm were not significantly different (intervention: mean 63, SD 6.9; control: mean 61.8, SD 6.6; *P*=.21). We observed a significant reduction in the headache burden only in the intervention arm at follow-up (3.5 points; *P*=.009). The reduction in the headache burden was not statistically different between groups (difference-in-differences estimate –1.89, 95% CI –5 to 1.31; *P*=.25). Similarly, secondary outcomes were not significantly different between groups. Only 11.32% (303/2677) of alerts were acted upon.

**Conclusions:**

The use of an interruptive electronic alert did not significantly improve headache outcomes. Low use of alerts by health care providers prompts future alterations of the alert and exploration of alternative approaches.

## Introduction

Headache disorders are a major public health concern, with migraine being the second most common cause of disability [1]. The frequency of headaches is nearly twice as common in women, adults aged 18‐44 years, people with low family income (<US $35,000), and Native Americans [2]. Patients with migraines and chronic headaches experience greater than normal use of emergency department (ED) services, with headaches accounting for approximately 3% of all ED visits annually [2]. Headaches also directly impact workplace productivity, with an economic impact estimated to be $19.3 billion in 2019 [3].

Headaches account for up to 30% of referrals from primary care to neurology. Of these, nearly 50% of patients are ultimately diagnosed with migraines, and the majority have never adequately trialed first-line therapy prior to referral [4-7]. Barriers to primary care management of headaches include time constraints, access challenges, and lack of expertise to accurately diagnose and give appropriate treatment [8,9]. These barriers result in high referrals to specialized care, an easier option than drug initiation titration, monitoring, and adjustments. This leads to prolonged wait times, resulting in patients often remaining unmanaged and seeking care in urgent or emergent settings for episodic management [10,11].

The Geisinger system was an early adopter of the electronic health record (EHR) system (beginning in 1996), with its EHR implemented across all sites of care. The use of clinician decision support tools in the form of alerts within the EHR has increased exponentially in recent years [12]. These alerts have been used across different disease states within primary care to provide useful information to clinicians, shape their behaviors, and positively impact patient safety and outcomes [12-14]. Considering the utility of alerts and the challenges described above, we instituted an EHR-based, clinician-facing, interruptive alert that provides real-time guidance on managing patients with headaches to improve headache care before neurology referral. This includes assistance in collecting information about headache characteristics, guidance on medication management, and the opportunity to e-consult with neurology providers. The purpose of this pragmatic randomized controlled trial was to evaluate the impact of the alert on the management of primary headache disorders in primary care settings. We hypothesized that an electronic alert for the management of headaches improves patient-reported headache burden at 6 months among patients with headache managed in primary care.

## Methods

### Setting

Geisinger is an integrated health care delivery system located in central and northeastern Pennsylvania, serving more than 1 million patients yearly. Geisinger maintains 44 primary care and over 100 specialty clinics, which include 8 neurology practices with 43 neurology physicians all using the same EHR system.

### Study Design

We conducted a prospective stratified cluster-randomized controlled trial across 38 primary care sites to assess the impact of an interruptive electronic alert on the management of primary headache disorders. Eligible patients were enrolled and followed for 6 months post index encounter to evaluate headache management-related outcomes. It was uploaded to ClinicalTrials.gov and can be found using the registration number NCT05067725. The trial followed CONSORT (Consolidated Standards of Reporting Trials) reporting guidelines for randomized trials. We have also reported the e-CONSORT per the journal requirements [15].

### Site Randomization and Patient Enrollment

In total, 38 of 44 primary care clinic sites were selected for randomization based on their ability to participate. The sites were first stratified into 9 possible blocks based on 2 criteria: geographical location (west, central, or northeast) and patient-to–health care provider ratio (low, moderate, or high). Note that 3 of the 9 possible blocks did not contain any sites, leaving only 6 blocks for this study. Within each block, simple randomization was then used to assign each site to either the intervention or control arm, using a 1:1 ratio. A Microsoft Excel 365 (Microsoft Corporation) random number generator was used to generate the simple randomization allocation sequence. Patients received the treatment to which their site had been assigned, making this a cluster-randomized study with sites as clusters (Multimedia Appendix 1).

### Patient Selection and Enrollment

Patients were eligible if (1) the criteria for the alert were met, which included an in-person or telemedicine encounter and a headache diagnosis or chief complaint of headache (Multimedia Appendix 2); (2) they were aged 18 years or older; and (3) postvisit criteria were met, which included a confirmed diagnosis of primary headache disorder by manual medical record review and a minimum baseline Headache Impact Test 6 (HIT-6) score of 50 or higher or headache frequency of 12 days or greater in the last 3 months. Clinical input and the scale guidance document were used to determine thresholds for HIT-6 scores and headache frequency [16]. Manual medical record reviews were conducted by study team members (HA, AP, and SP) under the guidance of the neurologist (SF) to confirm headache diagnosis. Types of headaches in the analytic sample included migraine, chronic daily, tension-type, cluster, or medication overuse headaches, collectively grouped as “headaches.” Patients were excluded if they had a serious systemic illness (eg, headaches due to hypertensive emergencies, hepatic or renal failure, or cardiac failure), secondary headaches (eg, concussion), were pregnant, or were actively followed with neurology [17]. Patients with serious systemic illness were excluded from our study as it would have been difficult to distinguish whether headaches observed among them were due to primary or secondary factors.

**Figure 1.**
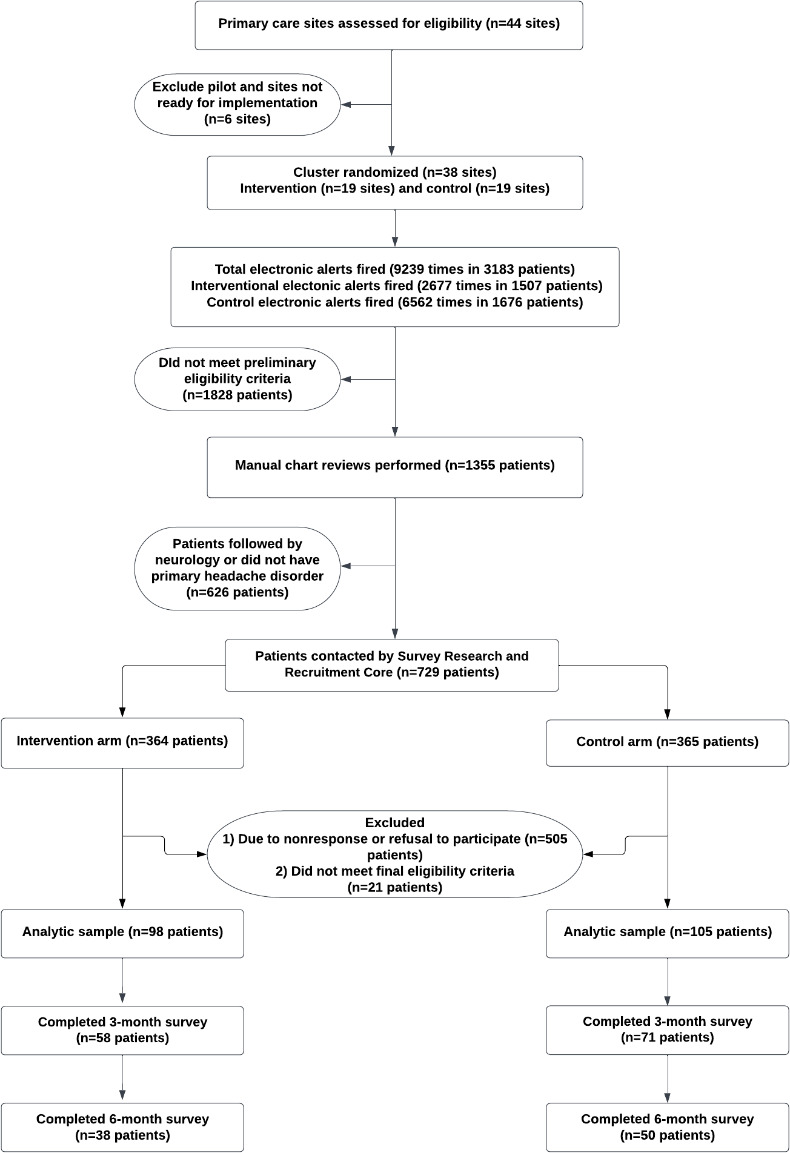
Patient selection, enrollment, and follow-up.

Within 2 weeks after their scheduled visit, eligible patients were contacted by the Survey Research and Recruitment Core to collect baseline measures (which determined minimum headache eligibility) and to gain verbal consent. Patient contact was attempted 7 times. Figure 1 represents this study’s process of patient selection, enrollment, and follow-up.

### Intervention

A clinician-facing interruptive EHR-based alert in the form of a Best Practice Advisory was built based on input from an expert panel of neurologists, primary care providers (PCPs), pharmacists, and informaticians. PCPs include primary care physicians and advanced practitioners such as nurse practitioners and physician assistants. The first iteration of the alert was developed based on input received from the needs assessment conducted with the PCPs. This version was piloted at 2 primary care sites over 3 months [18]. Face-to-face education of PCPs at the pilot sites was undertaken to familiarize health care providers with the utility of the alert [18]. A simple pre-post assessment of the 900 eligible patient encounters during this pilot phase demonstrated a significant reduction in neurology referrals post implementation of the electronic alert. Following the pilot implementation, feedback was collected from 10 PCPs at these sites about the structure, verbiage, and utility of the electronic alert. Subsequent modifications were made based on this feedback before full implementation.

The Best Practice Advisory consisted of four parts: (1) a questionnaire to assist the PCP in characterizing the patient’s headache (Multimedia Appendix 3); (2) a “SmartSet” guide for ordering medications, imaging, laboratories, and referrals using evidence-based guidelines (Multimedia Appendix 3); (3) a hyperlink to allow for electronic consultation with a neurologist; and (4) a link to the “synopsis” which provided a summary of prior patient headache care. The “SmartSet” guide could be accessed directly within the alert by clicking on the “Open Express Lane” button to facilitate quick ordering for health care providers. The alert appeared within the EHR when a PCP entered a headache diagnosis for an in-person or telemedicine encounter. All actions taken in the alert were recorded, although health care providers could dismiss them without any action. Suppression criteria were built to lock out and avoid repeated firing of the alert. The alert was suppressed for 24 hours if the dismissal reason was headache-controlled and for 365 days for PCP long-term management. The alert could also be dismissed by acknowledging that the health care provider was previewing the patients’ medical record, in this case, the alert would refire during the same encounter.

An invisible or silent alert was activated at control sites that would be triggered by identical criteria to the intervention arm, however, this alert was not visible to the clinicians, so no suppression criteria were developed for control sites. The silent alert was developed to help generate a list of eligible patients for the control group that would be comparable to those identified in the intervention arm.

Before full implementation, the primary care leadership team was encouraged to disseminate information about the tool to PCPs in the intervention cohort through local departmental meetings and direct emails, including text and short video instructions.

### Data Collection

Data sources included the EHR and survey results from patients at baseline, 3 months, and 6 months. The surveys conducted by the Survey Research and Recruitment Core included 6 questions from the HIT-6 questionnaire, which measures headache impact on a participant’s ability to function in day-to-day life [19]; 1 question on pain intensity; 1 on the frequency of headaches; 3 from the Migraine Treatment Optimization Questionnaire (M-TOQ), which determines if a medication treatment for a participant is optimal; and 2 asking for sociodemographic information that could not be pulled from the EHR [20]. The M-TOQ questions were modified for our study by replacing the word “migraine” with “headache.” Prior to study enrollment, the survey was piloted among a sample (n=10) of patients from pilot sites to ensure appropriateness and check for face validity. Patients had the option to opt-out at any time during this study. See Multimedia Appendix 4 for the full survey guide.

### Study Outcomes

#### Primary Outcome—Headache Burden

Headache burden was assessed using the HIT-6 scale [7]. The scale ranged from 36 to 78, and a score of 50 or greater represented a greater impact and disruption of life caused by headache [21,22]. The primary end point was the comparison of changes in HIT-6 scores between arms from baseline to 6 months.

#### Secondary Outcomes

Secondary outcomes included headache frequency, defined as the self-reported frequency of headache days over the previous 3 months; average headache pain intensity defined by pain on a 10-point analog scale over the same period (0=no pain at all to 10=the worst pain ever); access to care, defined as the proportion of patients referred to neurology; resource utilization, defined as the proportion of patients with an ED-visit (all-cause and headache specific); and medication management, defined as the proportion of patients that initiated preventive or abortive medications for headache [7,23]. Referral to neurology was used as a surrogate measure to represent access to neurological care considering that the availability of a timely neurology appointment impacts the volume of referrals.

#### Implementation Outcomes

We assessed the adoption of the alert among health care providers through actions within the alert. Positive adoption was any activity where the health care provider interacted with the components of the alert such as completing the headache questionnaire, using the SmartSet, or using the synopsis. We also reviewed feedback provided by PCPs from reports generated by a committee that oversees all EHR-based alerts.

Several modifications to the initial analysis plan were implemented following go-live but before analysis. We omitted some secondary outcomes, including time to initiation of treatment in treatment naïve patients and M-TOQ outcomes. The data on the M-TOQ were only available at baseline eliminating any pre-post analysis, and the time to initiation of medication in naïve patients was dropped in favor of initiation of treatment in all patients as a binary outcome. We added a referral to neurology due to its relevance to clinical practice. All these changes were updated on the ClinicalTrials.gov website to ensure reporting transparency. We also updated our analytical approach from generalized linear models with robust SEs in the analytical plan to generalized estimating equations (GEE), a specialized generalized linear model approach. After further internal discussion, we felt given that the outcomes data were longitudinal and correlated due to clustering, GEE were better suited.

### Sample Size Calculation

A minimum difference of equal to or greater than a 2.3-point (SD 4.3) reduction on the HIT-6 scale represents a clinically important and significant change [22]. We needed to enroll at least a total of 66 patients per arm to detect this change with 80% power at a significance level of .05 and an intraclass correlation of 0.02. The sample size was calculated using an online calculator developed by the University of California San Francisco [24].

### Statistical Analysis

The total number of patients in each arm was counted to ensure a balanced distribution of patient numbers. Descriptive statistics were computed for continuous variables as means or medians with SD or IQRs, and as frequency and percentages for categorical variables when appropriate for baseline patient-reported outcomes and socioeconomic and demographic characteristics (ie, sex, age, race, ethnicity, insurance, education, and income). Appropriate statistical tests were used to compare differences in these characteristics between the 2 groups and within groups: 2-tailed independent *t* tests or paired *t* tests were used for continuous data, *χ*^2^ for categorical data, and Mann-Whitney for nonnormal categorical or continuous data.

We constructed GEE for all outcome comparisons between groups, adjusting for effects of treatment, phase (baseline, 6 months), treatment-by-phase interaction, and demographics while accounting for clustering of patients per primary care site. The GEE model with log link was used, considering the skewness of data observed for headache days and pain scores (Multimedia Appendix 5). For all the binary variables, a log link with binomial distribution was used, while an identity link with normal distribution was used for HIT-6 scores. As a first step, we performed an unadjusted GEE model with this study’s groups as only a univariate analysis. Multivariable (adjusted) models only included demographic variables that were significantly different between groups (*P*<.05). For the GEE analysis, we reported odds ratios or estimates and associated CIs. All analyses were conducted using SAS Enterprise Guide software (version 8.3 for Windows; SAS Institute Inc).

The analysis was conducted based on the principle of “intention-to-treat (ITT)” analysis. All patients who consented and met the inclusion criteria were included in the analysis per the group they were recruited into. The analysis was performed to assess the difference in outcomes at the individual patient level. For missing data via survey collection, we applied a last observation carried forward (LOCF) technique to maximize the number of observations available for analysis [25]. The results from the LOCF technique were further compared to those from the measured data to assess the impact of loss to follow-up.

### Ethical Considerations

This study was an evaluation of a quality improvement initiative that was undertaken at Geisinger with the intent to affect clinical practice. As a result, it was determined as “not human subjects research” by Geisinger’s institutional review board (2021‐0729).

## Results

### Overview

The alert was fired for a total of 9239 times in 3183 patients (intervention arm: 2677 times in 1507 patients; control arm: 6562 times in 1676 patients) between December 2021 and February 2022. We excluded 1828 patients for not meeting eligibility criteria. We manually verified the diagnosis of primary headache among 729 of the remaining 1355 patients who were eligible for consent and baseline survey assessment. We obtained verbal consent from 221 patients at baseline but only enrolled the 203 patients that either had an HIT-6 score of ≥50 or a headache frequency of >12 days, as per our inclusion criteria (Figure 1). At the end of 6 months, follow-up patient-reported data were available for 88/203 (43.3%) patients.

All baseline characteristics were similar between groups for patients enrolled in this study except for mean age, which was higher in the intervention group (mean 43, SD 14.4 y vs mean 39, SD 14.4 y, *P*=.04; Table 1). There was no difference in the HIT-6 scores, headache frequency and intensity, or patient-reported use of medications at baseline between groups (HIT-6 scores: mean 63, SD 6.9 vs mean 61.8, SD 6.6; headache days in the past 3 months: median 20.5, IQR 10.0 - 45.0 vs median 20, IQR 10.0 - 45.0; pain scores: median 7, IQR 6.0 - 8.0 vs median 7, IQR 5.0 - 8.0; reported use of preventive or abortive medications: 63/98, 64% vs 57/105, 54.2%). The majority of the patients were female, were White, were non-Hispanic, had a commercial insurance plan, had an educational qualification of greater than high school, and had an annual household income between US $50,000 to US $100,000. Similarly, there was no statistically significant difference in the baseline characteristics observed between groups for patients that completed the 6-month follow-up.

**Table 1.** Patient characteristics at baseline.

Demographics		Intervention arm (n=98)	Control arm (n=105)	*P* value
Age (years), mean (SD)		43.2 (14.4)	39.1 (14.4)	.04
**Sex, n (%)**				.19
	Males	28 (28.6)	21 (20.0)	
	Females	70 (71.4)	84 (80.0)	
**Race, n (%)**				.56
	African American	2 (2.0)	2 (1.9)	
	White	95 (97)	98 (94.2)	
	Other	1 (1.0)	4 (3.8)	
**Ethnicity, n (%)**				.60
	Non-Hispanic	92 (93.9)	95 (91.4)	
	Hispanic	6 (6.1)	9 (8.6)	
**Type of insurance at the most recent encounter (members), n (%)**				.32
	Commercial	59 (60.2)	56 (53.3)	
	Medicare	13 (13.3)	12 (11.4)	
	Medicaid	26 (26.7)	31 (29.5)	
	Other	0 (0)	6 (5.8)	
**Highest level of education, n (%)**				.98
	<High school or GED^a^	9 (9.2)	9 (8.6)	
	High school or GED	32 (32.7)	38 (36.2)	
	Some college or technical program	29 (29.6)	26 (24.8)	
	4 y college (BS or BA)	16 (16.3)	20 (19.0)	
	Master’s degree (MS, MA, or MPH)	8 (8.2)	9 (8.6)	
	Doctorate (PhD or ScD or professional—MD, DO, or JD)	3 (3.0)	2 (1.9)	
	Refuse	1 (1.0)	1 (0.9)	
**Annual household income (US $), n (%)**				.12
	25,000 or less	20 (20.4)	26 (24.8)	
	Over 25,000-50,000	21 (21.4)	27 (25.7)	
	Over 50,000-100,000	34 (34.7)	21 (20.0)	
	Over 100,000	14 (14.3)	13 (12.4)	
	Refuse	9 (9.2)	18 (17.1)	
HIT-6^b^ scores, mean (SD)		63 (6.9)	61.8 (6.6)	.21
Number of headache days, median (IQR)		20.5 (10.0 - 45.0)	20 (10.0 - 45.0)	.87
Pain scores, median (IQR)		7 (6.0 - 8.0)	7 (5.0 - 8.0)	.91
Patient-reported use of preventive or abortive medications, n (%)		63 (64.3)	57 (54.2)	.14

a GED: General Educational Development.

b HIT-6: Headache Impact Test 6.

### Primary Outcome—Headache Burden

HIT-6 scores improved significantly from baseline in the intervention arm at 6 months (3.5 points; *P*=.009) but not in the control group (1.40 points; *P*=.23); additionally, the improvements from baseline did not differ significantly between groups (Table 2). The intracluster correlation coefficient observed was 0.411 for HIT-6 scores.

**Table 2.** Adjusted primary and secondary outcomes of headache burden, frequency, and intensity.

Outcome and arm		Phase	n	Mean (SD)	Change, mean (SD)	D-I-D^a^ estimate, regression coefficients (95% CI)	*P* value
**HIT-6^b^ score**						–1.84 (–5 to 1.31)	.25
	**Intervention**				–3.5 (7.9)^c^		
		Baseline	98	63.0 (6.9)			
		Month 6	38	58.7 (8.6)			
	**Control**				–1.40 (8.1)		
		Baseline	105	61.8 (6.5)			
		Month 6	50	60.1 (8.7)			
**Headache days**						1.22 (0.82 to 1.81)	.24
	**Intervention**				–9.2 (30.5)		
		Baseline	98	30.9 (25.8)			
		Month 6	38	22.8 (26.2)			
	**Control**				–9.3 (16.3)^c^		
		Baseline	105	28.9 (23.5)			
		Month 6	50	18.4 (18.2)			
**Pain**						1 (0.9 to 1.11)	.99
	**Intervention**				–0.395 (1.59)		
		Baseline	98	6.9 (1.8)			
		Month 6	38	6.5 (1.8)			
	**Control**				–0.340 (1.92)		
		Baseline	105	6.9 (1.9)			
		Month 6	50	6.4 (2.1)			

a D-I-D: difference-in-differences.

b HIT-6: Headache Impact Test 6.

c *P=*.01.

### Secondary Outcomes

Change in the number of headache days and pain score did not differ between groups (Table 2). Similarly, compared to baseline, there was no difference in the proportion of patients being referred to neurology, using ED for all-cause or headache-specific reasons, or initiating new abortive or preventative treatment in the 6 months post intervention (Table 3).

**Table 3.** Percent change in the proportion of patients from baseline to 6 months and adjusted odds ratio estimates for resource use and access to care (note: a negative or positive percentage denotes a decrease or increase post recruitment).

Outcome and arm		Phase	Use, n (%)	Percent change	D-I-D^a^ estimate, odds ratio (95% CI)	*P* value
**ED**^**b**^ **use (all-cause)**					1.4 (0.58 to 3.36)	.46
	**Intervention (n=98)**			–2.0		
		Baseline	19 (19.4)			
		Month 6	17 (17.4)			
	**Control (n=105)**			–4.8		
		Baseline	15 (14.3)			
		Month 6	10 (9.5)			
**Neurology referral**					0.52 (0.064 to 4.29)	.55
	**Intervention (n=98)**			12.3^c^		
		Baseline	2 (2.04)			
		Month 6	14 (14.3)			
	**Control (n=105)**			21.0^d^		
		Baseline	2 (1.9)			
		Month 6	24 (22.9)			
**Medication initiation**					0.91 (0.51 to 1.60)	.73
	**Intervention (n=98)**			25.5^d^		
		Baseline	50 (51.0)			
		Month 6	75 (76.5)			
	**Control (n=105)**			28.5^d^		
		Baseline	49 (46.7)			
		Month 6	79 (75.2)			
**ED use (headache-specific)**					0.49 (0.051 to 4.72)	.54
	**Intervention (n=98)**			–2.10		
		Baseline	4 (4.1)			
		Month 6	2 (2.0)			
	**Control (n=105)**			0.0		
		Baseline	4 (3.81)			
		Month 6	4 (3.81)			

a D-I-D: difference-in-differences.

b ED: emergency department

c *P*=.002.

d *P*<.001.

Results from the LOCF models for HIT-6 scores, headache days, and pain intensity are listed in Table 4. Findings from the LOCF models were similar to those from the base models presented in Table 2. It was observed that there was a similar significant improvement in HIT-6 scores in the intervention arm, and a reduction in the headache days in the control arm from baseline to 6 months, however, the difference between groups was not statistically significant.

**Table 4.** Adjusted primary and secondary outcomes of headache burden, frequency, and intensity using the LOCF^a^ method.

Outcome and arm		Phase	N	Mean (SD)	Change, mean (SD)	D-I-D^b^ estimate, regression coefficients (95% CI)	*P* value
**HIT-6 score**						–1.01 (–2.87 to 0.85)	.29
	**Intervention**				–1.36 (5.2)^c^		
		Baseline	98	63 (6.9)			
		Month 6	98	61 (8.2)			
	**Control**				–0.67 (5.6)		
		Baseline	105	61.8 (6.5)			
		Month 6	105	60.8 (8.4)			
**Headache days**						1.07 (0.87 to 1.32)	.49
	**Intervention**				–3.57 (19.3)		
		Baseline	98	30.9 (25.8)			
		Month 6	98	27.5 (22.9)			
	**Control**				–4.41 (17.6)^c^		
		Baseline	105	28.9 (23.5)			
		Month 6	105	24 (22.6)			
**Pain**						1.02 (0.95 to 1.10)	.60
	**Intervention**				–0.153 (1.00)		
		Baseline	98	6.9 (1.82)			
		Month 6	98	6.6 (2.01)			
	**Control**				–0.162 (1.30)		
		Baseline	105	6.9 (1.9)			
		Month 6	105	6.4 (2.1)			

a LOCF: last observation carried forward.

b D-I-D: difference-in-differences.

c *P=*.01.

### Implementation Outcomes

Of the 2677 alert firings in the intervention arm, 2228 (83.23%) were overridden or dismissed, 146 (5.45%) were ignored, and 303 (11.32%) action was taken. Of the 205 PCPs exposed to the intervention alert, 15 provided feedback, 3 found the alert helpful, and 12 expressed dissatisfaction due to alert firing on inappropriate patients or noting the alert was disruptive to workflow. A manual medical record review of 1355 patients revealed that 729 (53.80%) had a confirmed diagnosis of primary headache disorder. The remaining 626 (46.20%) either did not have a confirmed diagnosis of primary headache disorder or were already followed by neurology for headaches.

## Discussion

In this pragmatic clinical trial across 38 clinics, we found no benefit of the electronic alert as designed on headache outcomes. As the alert was based on evidence-based guidelines for diagnosis and treatment, it seems unlikely that the content of the alert was poor and more likely that the lack of impact was due to gaps in implementation.

This is the first study to offer a clinician decision support tool that guided PCPs in diagnosing and appropriately treating patients with different types of primary headache disorders using an episodic electronic alert. Most of the research studies to date focus on a single type of headache disorder, chronic headaches, or migraines [23,26]. As initial preventative treatment for the most common headache types is similar, our alert was designed to be used across them as a one-stop access to help diagnose and treat headaches and e-consult with neurologists if required.

Even though we did notice improvements in several outcome measures in both the intervention and control arms from baseline to 6-month follow-up, on a between-groups comparison, the effects did not hold. A similar trend for improvement from baseline in outcomes such as HIT-6 scores and headache days was observed when an LOCF approach was used, but the effect did not hold for between-group comparisons. Using our pragmatic design, we were able to distinguish between expected improvements in headaches over time and those attributed to the intervention using a comparator group, with largely similar characteristics except by age at baseline [27]. Past research studies that have evaluated the impact of headache management programs and found improvement in patients’ headache burden and resource use have often used a pre-post study design which makes attribution of success to the intervention difficult [17,23]. Our results have substantial implications, as identification of the ineffectiveness of these types of alerts is necessary to determine which should remain active and which should be either deactivated or revised. Only a well-designed test of comparison with a suitable counter-factual arm (ie, control) can distinguish improvements due to bias (eg, regression to the mean) versus the intervention itself.

The total number of unique patients for whom the alert fired was relatively similar between groups, but the alert fired 2.2 times more frequently in the control arm. This is to be expected, as in the interventional arm, the interruptive alert that was visible to the health care providers could be suppressed for the duration of the encounter by acknowledging a reason for dismissal, as opposed to the silent or invisible alert in the control arm. Health care providers also noted several challenges with alert firing such as confusion with multiple components of the alert, concerns over alert firing for inappropriate patients and appearing at times other than during direct patient contact, and trouble dismissing the alert, highlighting flaws with the existing alert design. Medical record reviews illustrated that health care providers often list headaches as not just chief complaints but also visit diagnoses, due to headaches often being a symptom of other underlying conditions. The inaccurate use of headache as a visit diagnosis led to the alert’s misfiring in nearly 40% of our patients. It also complicates the firing logic for decision support tools such as ours. Research shows that the nonspecificity of alerts may desensitize clinicians and lead to habituation, thereby lessening their likelihood to follow alert recommendations or guidance [28,29]. Furthermore, even when firing appropriately, health care providers reported the location of the alert to be disruptive to their normal workflow. Some of the challenges mentioned could be attributed to the health care providers not having received sufficient education about the alert. The majority of the health care providers did not recall having seen or received any education about the alert despite efforts to disseminate this information through emails, fact sheets, and videos. This highlights an implementation gap and the need for a more engaged educational outreach in primary care settings.

Much of the feedback and low adoption found in our study affirms prior reports of alert fatigue experienced by health care providers in primary care settings and is likely to be the main contributor to the lack of significant impact [30]. With only 11.32% (303/2677) of alerts being acted upon, we can expect only this fraction to benefit from the intervention. Yet, research does not support the notion that prior alert exposure has a carry-forward impact on the care of future patients; suggesting that future recognition of patients and bypassing of alerts does not have a strong persistence of effect on health care provider action behaviors [28,29,31].

The lack of positive outcomes within the intervention arm was unexpected, considering the detailed human-centered design-build, the engagement of pilot PCPs, and the early feedback received, which was affirmative for the intervention. Slight modifications to the verbiage within the alert were undertaken based on feedback from the pilot health care providers before its full-scale implementation. Despite this, we noted low use of the features present within the alert and additional adoption challenges that were not fully uncovered in the pilot period. These results are similar to other studies published using human-centered design, which failed to translate into adoption and outcome differences [32-35]. These studies have found that better engagement by pilot health care providers does not always translate to all health care providers at the time of scaling interventions. Additionally, differences in the mechanism used for delivering training between the pilot and full implementation phase also impact the uptake of the intervention [32-35]. Furthermore, the positive findings and feedback from the pilot phase could be influenced by commitment and congruence bias in addition to observation and measurement bias resulting in a Hawthorne effect of the intervention [36].

Considering the challenges identified in this study, post completion we made significant modifications to the tool. First, we redesigned the identification of the patients to make it more specific (but less sensitive) to the targeted patient population. We repositioned the alert to fire at the time of neurology referral as opposed to when assigning a visit diagnosis or chief complaint. We also recognize that an EHR-only intervention is not the only mechanism to address incomplete headache care, and a pilot initiative involving pharmacist-based medication management has been launched. Research is currently underway to assess the impact of this pilot.

We recognize several limitations to our study. First, given the single-site design of this study, generalizability to other sites is limited and likely influenced by variations in practice environments and standards of practice. Our use of an alert was largely driven by our integrative health care environment, the ubiquitous use of EHR across primary care and specialty health care providers, and the low cost and light maintenance of the alert. Second, while an LOCF method was used to reduce the effect of a type II error, the high attrition rate observed might have affected the magnitude of the impact. We recognize that other interventions to address the optimal treatment of headaches do exist and are worthy of consideration. Programs that involved greater manpower and dedicated professionals for follow-up and management observed significant improvement in their patients’ headache burden and headache-related disability or reduced resource use, and attributed their success to the dedicated personnel [23,26]. Alternatively, programs that solely relied on physician education or leveraging existing clinical pathways, while at times cost-saving, did not significantly improve patients’ headache burden or quality of life [7,21]. Most of these existing studies have been standalone initiatives that focused on headache-specific work groups with limited generalizability of results, some of which are no longer in effect due to resource and infrastructure constraints. Our objective was to sustainably change the culture of practice surrounding headache care without creating new standalone avenues. In conclusion, our study found that the implementation of an interruptive electronic alert in primary care to aid diagnosis and management of primary headache disorders did not improve headache care for patients. Low adoption of the tool by the health care providers has prompted the development of alternative population health–based approaches to improve headache management.

## Supplementary material

10.2196/58456 Multimedia Appendix 1 Site-level distribution based on stratification.

10.2196/58456 Multimedia Appendix 2 Project inclusion and exclusion *ICD* codes. *ICD*: *International Classification of Diseases*.

10.2196/58456 Multimedia Appendix 3 Snapshots of the electronic alert questionnaire and Express Lane or SmartSet tool.

10.2196/58456 Multimedia Appendix 4 Survey administered by Geisinger’s SRRC. SRRC: Survey Research and Recruitment Core.

10.2196/58456 Multimedia Appendix 5 Distribution of pain scores and the number of headache days observed in the intervention and control arm of this study.

10.2196/58456 Checklist 1 CONSORT-eHEALTH checklist (V 1.6.1).

## Abbreviations

CONSORT: Consolidated Standards of Reporting Trials
ED: emergency department
EHR: electronic health record
GEE: generalized estimating equations
HIT-6: Headache Impact Test 6
ITT: intention-to-treat
LOCF: last observation carried forward
M-TOQ: Migraine Treatment Optimization Questionnaire
PCP: primary care provider
