# Supplementary material for: Impact of an Electronic Health Record–Based Interruptive Alert Among Patients With Headaches Seen in Primary Care: Cluster Randomized Controlled Trial
Source: JMIR Med Inform. 2024 Aug 29;12:e58456. doi: 10.2196/58456 (PMC11376138; doi:10.2196/58456)
Supplement: Multimedia Appendix 1 [file medinform-v12-e58456-s001.docx]

**Multimedia Appendix 1.** Site-level distribution based on stratification.

| **Strata** | **Number of Control clinic sites (N=19 sites)** | **Number of Intervention clinic sites (N=19 sites)** |
| --- | --- | --- |
| West Low | 5 | 5 |
| West High | 1 | 1 |
| Northeast Low | 4 | 4 |
| Northeast Moderate | 2 | 3 |
| Central Low | 5 | 4 |
| Central Moderate | 2 | 2 |
