# Supplementary material for: Impact of an Electronic Health Record–Based Interruptive Alert Among Patients With Headaches Seen in Primary Care: Cluster Randomized Controlled Trial
Source: JMIR Med Inform. 2024 Aug 29;12:e58456. doi: 10.2196/58456 (PMC11376138; doi:10.2196/58456)
Supplement: Multimedia Appendix 2 [file medinform-v12-e58456-s002.docx]

**Multimedia Appendix 2.** Project inclusion and exclusion ICD codes.

***Inclusion Criteria***

| **Name** | **External ID** | **Id** | **ICD-9-CM** | **ICD-10-CM** |
| --- | --- | --- | --- | --- |
| Tension headache | 103268 | 3268 | 307.81 |  |
| Migraine with aura | 103583 | 3583 | 346 | G43.109 |
| Migraine with aura, intractable | 103584 | 3584 | 346.01 | G43.119 |
| Migraine without aura | 103586 | 3586 | 346.1 | G43.009 |
| Migraine without aura, intractable | 103587 | 3587 | 346.11 | G43.019 |
| Migraine variant | 103589 | 3589 | 346.2 | G43.809 |
| Migraine variant, intractable | 103590 | 3590 | 346.21 | G43.819 |
| Migraine | 103595 | 3595 | 346.9 | G43.909 |
| Lumbar puncture reaction | 103608 | 3608 | 349 | G97.1 |
| Post-dural puncture headache | 116363 | 16363 | 349 | G97.1 |
| Sleep related headaches | 116936 | 16936 | 784 | R51.9 |
| Cluster headaches | 117627 | 17627 | 339 | G44.009 |
| Cluster headache, episodic | 117628 | 17628 | 339.01 | G44.019 |
| Cluster headache, chronic | 117629 | 17629 | 339.02 | G44.029 |
| Paroxysmal hemicrania, chronic | 117631 | 17631 | 339.04 | G44.049 |
| SUNCT (short unilateral neuralgiform headache, conjunctival inj/tear) | 117632 | 17632 | 339.05 | G44.059 |
| Tension type headache | 117634 | 17634 | 339.1 | G44.209 |
| Episodic tension-type headache | 117635 | 17635 | 339.11 | G44.219 |
| Chronic tension-type headache | 117636 | 17636 | 339.12 | G44.229 |
| Headache, hypnic | 117645 | 17645 | 339.81 | G44.81 |
| Headache, primary cough | 117647 | 17647 | 339.83 | G44.83 |
| Headache, primary exertional | 117648 | 17648 | 339.84 | G44.84 |
| Headache, primary stabbing | 117649 | 17649 | 339.85 | G44.85 |
| Migraine with aura with status migrainosus | 117651 | 17651 | 346.02 | G43.101 |
| Migraine with aura, intractable, with status migrainosus | 117652 | 17652 | 346.03 | G43.111 |
| Migraine without aura with status migrainosus | 117653 | 17653 | 346.12 | G43.001 |
| Migraine without aura, intractable, with status migrainosus | 117654 | 17654 | 346.13 | G43.011 |
| Migraine variant with status migrainosus | 117655 | 17655 | 346.22 | G43.801 |
| Migraine, hemiplegic | 117657 | 17657 | 346.3 | G43.409 |
| Migraine, hemiplegic, intractable | 117658 | 17658 | 346.31 | G43.419 |
| Migraine, hemiplegic, with status migrainosus | 117659 | 17659 | 346.32 | G43.401 |
| Migraine, hemiplegic, intractable, with status migrainosus | 117660 | 17660 | 346.33 | G43.411 |
| Migraine, menstrual | 117661 | 17661 | 346.4 | G43.829 |
| Migraine, menstrual, intractable | 117662 | 17662 | 346.41 | G43.839 |
| Migraine, menstrual, with status migrainosus | 117663 | 17663 | 346.42 | G43.821 |
| Migraine, menstrual, intractable, with status migrainosus | 117664 | 17664 | 346.43 | G43.831 |
| Migraine aura, persistent | 117665 | 17665 | 346.5 | G43.509 |
| Migraine aura, persistent, intractable | 117666 | 17666 | 346.51 | G43.519 |
| Migraine aura, persistent, with status migrainosus | 117667 | 17667 | 346.52 | G43.501 |
| Migraine aura, persistent, intractable, with status migrainosus | 117668 | 17668 | 346.53 | G43.511 |
| Migraine aura, persistent, with cerebral infarction (HCC) | 117669 | 17669 | 346.60, 434.91 | G43.609, I63.9 |
| Migraine aura, persistent, with cerebral infarct, intractable (HCC) | 117670 | 17670 | 346.61, 434.91 | G43.619, I63.9 |
| Migraine aura, persistent, with cerebral infarct, status over 72 hours | 117671 | 17671 | 346.62, 434.91 | G43.601, I63.9 |
| Migraine aura, persistent, w/cerebral infarct, intract, status >72hrs | 117672 | 17672 | 346.63, 434.91 | G43.611, I63.9 |
| Migraine, chronic, without aura | 117673 | 17673 | 346.7 | G43.709 |
| Migraine, chronic, without aura, intractable | 117674 | 17674 | 346.71 | G43.719 |
| Migraine, chronic, without aura, with status migrainosus | 117675 | 17675 | 346.72 | G43.701 |
| Migraine, chronic, without aura, intractable, with status migrainosus | 117676 | 17676 | 346.73 | G43.711 |
| Migraine with status migrainosus | 117679 | 17679 | 346.92 | G43.901 |
| Migraine with intractable migraine, so stated, with status migrainosus | 117680 | 17680 | 346.93 | G43.911 |
| Reaction to spinal puncture | 130674 | 30674 | 349 | G97.1 |
| Reaction to lumbar puncture | 130675 | 30675 | 349 | G97.1 |
| Headache following lumbar puncture | 130676 | 30676 | 349 | G97.1 |
| Classical migraine | 130984 | 30984 | 346 | G43.109 |
| Common migraine | 130985 | 30985 | 346.1 | G43.009 |
| Atypical migraine | 130986 | 30986 | 346.1 |  |
| Sick headache | 130987 | 30987 | 346.1 |  |
| Abdominal migraine | 130988 | 30988 | 346.2 | G43.D0 |
| Decapitated migraine | 130989 | 30989 | 346.2 | G43.D0 |
| Classical migraine with intractable migraine, so stated | 139342 | 39342 | 346.01 | G43.119 |
| Common migraine with intractable migraine, so stated | 139343 | 39343 | 346.11 | G43.019 |
| Variants of migraine | 139344 | 39344 | 346.2 | G43.809 |
| Variants of migraine with intractable migraine, so stated | 139345 | 39345 | 346.21 | G43.819 |
| Other forms of migraine | 139346 | 39346 | 346.8 |  |
| Migraine, unspecified | 139348 | 39348 | 346.9 |  |
| Facial pain | 147302 | 47302 | 784 | R51.9 |
| Facial pains | 147303 | 47303 | 784 |  |
| Pain, facial | 147304 | 47304 | 784 |  |
| Pains, facial | 147305 | 47305 | 784 |  |
| Cephalalgia | 148849 | 48849 | 784 | R51.9 |
| Cephalgia | 148850 | 48850 | 784 | R51.9 |
| Cephalodynia | 148851 | 48851 | 784 | R51.9 |
| Head ache | 148852 | 48852 | 784 | R51.9 |
| Head pain | 148853 | 48853 | 784 | R51.9 |
| Head pain cephalgia | 148854 | 48854 | 784 | R51.9 |
| Headaches | 148855 | 48855 | 784 |  |
| Pain head | 148856 | 48856 | 784 | R51.9 |
| Pain, head | 148857 | 48857 | 784 | R51.9 |
| Pains, head | 148858 | 48858 | 784 |  |
| Headache, migraine | 149151 | 49151 | 346.9 | G43.909 |
| Migraine headache | 149152 | 49152 | 346.9 | G43.909 |
| Migraines | 149153 | 49153 | 346.9 | G43.909 |
| Headache, psychogenic | 154921 | 54921 | 307.81 |  |
| Headache, tension | 154922 | 54922 | 307.81 |  |
| Headache, tension-type | 154923 | 54923 | 307.81 |  |
| Headaches, tension | 154924 | 54924 | 307.81 |  |
| Headaches, tension-type | 154925 | 54925 | 307.81 |  |
| Muscle tension headache | 154926 | 54926 | 307.81 | G44.209 |
| Psychogenic cephalalgia | 154927 | 54927 | 307.81 | F45.41 |
| Psychogenic headache | 154928 | 54928 | 307.81 | F45.41 |
| Tension headaches | 154929 | 54929 | 307.81 |  |
| Tension type headache | 154930 | 54930 | 307.81 |  |
| Tension, headache | 154931 | 54931 | 307.81 |  |
| Tension-type headaches | 154932 | 54932 | 307.81 |  |
| Headache vascular | 158133 | 58133 | 784 |  |
| Headache, vascular | 158134 | 58134 | 784 |  |
| Headaches, vascular | 158135 | 58135 | 784 |  |
| Vascular headache | 158136 | 58136 | 784 | G44.1 |
| Vascular headaches | 158137 | 58137 | 784 |  |
| Migraine syndrome | 159792 | 59792 | 346 | G43.909 |
| Syndrome, migraine | 159793 | 59793 | 346 |  |
| Headache, sick | 160375 | 60375 | 346.1 |  |
| Migraine, common | 160376 | 60376 | 346.1 |  |
| Classic migraine | 161430 | 61430 | 346 | G43.109 |
| Migraine, classical | 161431 | 61431 | 346 |  |
| Migraine, abdominal | 168922 | 68922 | 346.2 | G43.D0 |
| Pain in head | 174259 | 74259 | 784 | R51.9 |
| Classical migraine without mention of intractable migraine | 176009 | 76009 | 346 | G43.109 |
| Common migraine without mention of intractable migraine | 176065 | 76065 | 346.1 | G43.009 |
| Other forms of migraine without mention of intractable migraine | 176766 | 76766 | 346.8 |  |
| Unspecified migraine without mention of intractable migraine | 177513 | 77513 | 346.9 |  |
| Variants of migraine without mention of intractable migraine | 177721 | 77721 | 346.2 | G43.809 |
| Classical migraine/not intrc | 183950 | 83950 | 346 |  |
| Common migraine/not intrc | 183952 | 83952 | 346.1 |  |
| Migrain vrient/not intrc | 183954 | 83954 | 346.2 |  |
| Classical migraine with intractable migraine | 190945 | 90945 | 346.01 | G43.119 |
| Common migraine with intractable migraine | 190946 | 90946 | 346.11 | G43.019 |
| Variants of migraine with intractable migraine | 190947 | 90947 | 346.21 | G43.819 |
| Migraine | 193046 | 93046 | 346.8 |  |
| Autonomic facial cephalgia | 195086 | 95086 | 337.9, 784.0 | G90.8, R51.9 |
| Chronic headache | 195271 | 95271 | 784 | R51.9, G89.29 |
| Head holding | 195428 | 95428 | 784 |  |
| Cephalalgias | 197027 | 97027 | 784 |  |
| Cephalgias | 197028 | 97028 | 784 |  |
| HA (headache) | 198948 | 98948 | 784 | R51.9 |
| Headache, cephalalgia | 199048 | 99048 | 784 |  |
| Lumbar puncture headache | 200032 | 100032 | 349 | G97.1 |
| Lumbar puncture react. | 200033 | 100033 | 349 |  |
| Post lumbar puncture headache | 201537 | 101537 | 349 | G97.1 |
| Postmyelography headache | 201560 | 101560 | 349 | G97.1 |
| Postspinal headache | 201564 | 101564 | 349 | G97.1 |
| Spinal headache | 202292 | 102292 | 349 | G97.1 |
| Stress headache | 202394 | 102394 | 307.81 | F45.41 |
| Migraine headaches | 211498 | 111498 | 346.9 |  |
| Migraine variants | 211499 | 111499 | 346.2 |  |
| Migraine, atypical | 211500 | 111500 | 346.1 |  |
| Migraine, variant | 211501 | 111501 | 346.2 |  |
| Migraine, with aura | 211502 | 111502 | 346 |  |
| Pain face | 212564 | 112564 | 784 |  |
| Pain in face | 212566 | 112566 | 784 | R51.9 |
| Pain, face | 212580 | 112580 | 784 | R51.9 |
| Pains, face | 212584 | 112584 | 784 | R51.9 |
| Migraine headaches | 217401 | 117401 | 346.8 |  |
| Common migraines | 218594 | 118594 | 346.1 |  |
| Migraines, common | 218596 | 118596 | 346.1 |  |
| Face pain | 219241 | 119241 | 784 | R51.9 |
| Facial pain, referred | 219242 | 119242 | 784 |  |
| Craniofacial pain | 219243 | 119243 | 784 | R51.9 |
| Pain, craniofacial | 219244 | 119244 | 784 |  |
| Pain, referred facial | 219245 | 119245 | 784 |  |
| Orofacial pain | 219246 | 119246 | 784 |  |
| Pain, orofacial | 219247 | 119247 | 784 |  |
| Vertex headaches | 219608 | 119608 | 784 |  |
| Vertex headache | 219609 | 119609 | 784 | R51.9 |
| Unilateral headaches | 219610 | 119610 | 784 |  |
| Unilateral headache | 219611 | 119611 | 784 | R51.9 |
| Headaches, thunderclap | 219612 | 119612 | 784 |  |
| Headache, thunderclap | 219613 | 119613 | 784 |  |
| Headache, unilateral | 219614 | 119614 | 784 |  |
| Headache, vertex | 219615 | 119615 | 784 |  |
| Headaches, bilateral | 219616 | 119616 | 784 |  |
| Headaches, generalized | 219617 | 119617 | 784 |  |
| Bilateral headaches | 219618 | 119618 | 784 | R51.9 |
| Headaches, unilateral | 219619 | 119619 | 784 |  |
| Headaches, ocular | 219620 | 119620 | 784 |  |
| Cephalodynias | 219621 | 119621 | 784 |  |
| Headaches, throbbing | 219622 | 119622 | 784 |  |
| Headaches, sharp | 219623 | 119623 | 784 |  |
| Headaches, retro-ocular | 219624 | 119624 | 784 |  |
| Headaches, orthostatic | 219625 | 119625 | 784 |  |
| Bilateral headache | 219626 | 119626 | 784 | R51.9 |
| Cranial pains | 219627 | 119627 | 784 |  |
| Headache, bilateral | 219628 | 119628 | 784 |  |
| Head pains | 219629 | 119629 | 784 |  |
| Generalized headache | 219630 | 119630 | 784 | R51.9 |
| Headaches, periorbital | 219631 | 119631 | 784 |  |
| Headache, generalized | 219632 | 119632 | 784 |  |
| Generalized headaches | 219633 | 119633 | 784 | R51.9 |
| Headache, throbbing | 219634 | 119634 | 784 |  |
| Headaches, vertex | 219635 | 119635 | 784 |  |
| Cranial pain | 219636 | 119636 | 784 | R51.9 |
| Retro ocular headache | 219637 | 119637 | 784 |  |
| Periorbital headache | 219638 | 119638 | 784 | R51.9 |
| Periorbital headaches | 219639 | 119639 | 784 |  |
| Retro-ocular headaches | 219640 | 119640 | 784 |  |
| Ocular headache | 219641 | 119641 | 784 | R51.9 |
| Thunderclap headache | 219642 | 119642 | 784 | G44.53 |
| Throbbing headaches | 219643 | 119643 | 784 |  |
| Throbbing headache | 219644 | 119644 | 784 | R51.9 |
| Pains, cranial | 219645 | 119645 | 784 |  |
| Pain, cranial | 219646 | 119646 | 784 |  |
| Thunderclap headaches | 219647 | 119647 | 784 |  |
| Orthostatic headache | 219648 | 119648 | 784 | R51.0 |
| Ocular headaches | 219649 | 119649 | 784 |  |
| Orthostatic headaches | 219650 | 119650 | 784 |  |
| Sharp headaches | 219651 | 119651 | 784 |  |
| Sharp headache | 219652 | 119652 | 784 | R51.9 |
| Acute confusional migraine | 220382 | 120382 | 346.9 | G43.909 |
| Acute confusional migraine | 220383 | 120383 | 346.8 |  |
| Headaches, migraine | 220385 | 120385 | 346.9 |  |
| Headaches, migraine | 220387 | 120387 | 346.8 |  |
| Complicated migraines | 220390 | 120390 | 346.8 |  |
| Confusional migraine, acute | 220392 | 120392 | 346.9 |  |
| Confusional migraine, acute | 220394 | 120394 | 346.8 |  |
| Confusional migraines, acute | 220396 | 120396 | 346.9 |  |
| Confusional migraines, acute | 220398 | 120398 | 346.8 |  |
| Acute confusional migraines | 220400 | 120400 | 346.9 |  |
| Acute confusional migraines | 220401 | 120401 | 346.8 |  |
| Complicated migraine | 220404 | 120404 | 346.8 |  |
| Migraine, complicated | 220407 | 120407 | 346.8 |  |
| Migraine, hemicrania | 220409 | 120409 | 346.9 |  |
| Migraine, hemicrania | 220411 | 120411 | 346.8 |  |
| Migraine, acute confusional | 220413 | 120413 | 346.9 |  |
| Migraine, acute confusional | 220415 | 120415 | 346.8 |  |
| Migraines, complicated | 220418 | 120418 | 346.8 |  |
| Migraines, acute confusional | 220420 | 120420 | 346.9 |  |
| Migraines, acute confusional | 220422 | 120422 | 346.8 |  |
| Migraines, hemicrania | 220424 | 120424 | 346.9 |  |
| Migraines, hemicrania | 220426 | 120426 | 346.8 |  |
| Headaches, tension-vascular | 222176 | 122176 | 307.81 |  |
| Headaches, stress | 222177 | 122177 | 307.81 |  |
| Headache, tension-vascular | 222178 | 122178 | 307.81 |  |
| Headaches, psychogenic | 222179 | 122179 | 307.81 |  |
| Psychogenic headaches | 222180 | 122180 | 307.81 |  |
| Tension-vascular headache | 222181 | 122181 | 307.81 |  |
| Tension-vascular headaches | 222182 | 122182 | 307.81 |  |
| Stress headaches | 222183 | 122183 | 307.81 | F45.41 |
| Tension vascular headache | 222184 | 122184 | 307.81 | G44.209 |
| Vascular cephalgia | 222452 | 122452 | 784 |  |
| Cephalgia, vascular | 222453 | 122453 | 784 |  |
| Cephalgias, vascular | 222454 | 122454 | 784 |  |
| Bad headache | 223281 | 123281 | 784 | R51.9 |
| Severe headache | 223351 | 123351 | 784 | R51.9 |
| Headache, migraine (classical) | 223984 | 123984 | 346 |  |
| Headache, migraine (common) | 223985 | 123985 | 346.1 |  |
| Migraine headache, classical | 224190 | 124190 | 346 |  |
| Migraine headache, common | 224191 | 124191 | 346.1 |  |
| Pain in the face | 224271 | 124271 | 784 |  |
| Pain in the head | 224274 | 124274 | 784 | R51.9 |
| Acute onset aura migraine | 224974 | 124974 | 346 | G43.109 |
| Headache, stress | 226155 | 126155 | 307.81 |  |
| Referred facial pain | 227362 | 127362 | 784 | R51.9 |
| Vascular cephalgias | 227971 | 127971 | 784 |  |
| Basilar migraines | 229478 | 129478 | 346 |  |
| Classic migraines | 229479 | 129479 | 346 |  |
| Classical migraines | 229480 | 129480 | 346 |  |
| Familial hemiplegic migraine | 229481 | 129481 | 346 |  |
| Familial hemiplegic migraines | 229482 | 129482 | 346 |  |
| Hemiplegic migraine, familial | 229483 | 129483 | 346 |  |
| Hemiplegic migraines, familial | 229484 | 129484 | 346 |  |
| Migraine aura without headache | 229485 | 129485 | 346 | G43.109 |
| Migraine with acute onset aura | 229486 | 129486 | 346 | G43.109 |
| Migraine with auras | 229487 | 129487 | 346 |  |
| Migraine with prolonged aura | 229488 | 129488 | 346 | G43.109 |
| Migraine with typical aura | 229489 | 129489 | 346 | G43.109 |
| Migraine, classic | 229490 | 129490 | 346 |  |
| Migraine, familial hemiplegic | 229491 | 129491 | 346 |  |
| Migraine, prolonged aura | 229492 | 129492 | 346 |  |
| Migraines, basilar | 229493 | 129493 | 346 |  |
| Migraines, classic | 229494 | 129494 | 346 |  |
| Migraines, classical | 229495 | 129495 | 346 |  |
| Migraines, familial hemiplegic | 229496 | 129496 | 346 |  |
| Migraines, prolonged aura | 229497 | 129497 | 346 |  |
| Prolonged aura migraine | 229498 | 129498 | 346 | G43.109 |
| Prolonged aura migraines | 229499 | 129499 | 346 |  |
| Acute onset aura migraine | 229500 | 129500 | 346.2 |  |
| Classic migraines | 229501 | 129501 | 346.2 |  |
| Classical migraines | 229502 | 129502 | 346.2 |  |
| Horton's syndrome | 229503 | 129503 | 346.2 |  |
| Migraine aura without headache | 229504 | 129504 | 346.2 |  |
| Migraine with acute onset aura | 229505 | 129505 | 346.2 |  |
| Migraine with auras | 229506 | 129506 | 346.2 |  |
| Migraine with prolonged aura | 229507 | 129507 | 346.2 |  |
| Migraine with typical aura | 229508 | 129508 | 346.2 |  |
| Migraine, classic | 229509 | 129509 | 346.2 |  |
| Migraine, prolonged aura | 229510 | 129510 | 346.2 |  |
| Migraines, classic | 229511 | 129511 | 346.2 |  |
| Migraines, classical | 229512 | 129512 | 346.2 |  |
| Migraines, prolonged aura | 229513 | 129513 | 346.2 |  |
| Prolonged aura migraine | 229514 | 129514 | 346.2 |  |
| Prolonged aura migraines | 229515 | 129515 | 346.2 |  |
| Syndrome, Horton | 229516 | 129516 | 346.2 |  |
| Syndrome, Horton's | 229517 | 129517 | 346.2 |  |
| Headache, ocular | 229540 | 129540 | 349 |  |
| Headache, orthostatic | 229541 | 129541 | 349 |  |
| Headache, periorbital | 229542 | 129542 | 349 |  |
| Headache, post-dural puncture | 229543 | 129543 | 349 |  |
| Headache, post-lumbar puncture | 229544 | 129544 | 349 | G97.1 |
| Headache, postdural puncture | 229545 | 129545 | 349 |  |
| Headache, retro-ocular | 229546 | 129546 | 349 |  |
| Headache, sharp | 229547 | 129547 | 349 |  |
| Headache, stabbing | 229548 | 129548 | 349 |  |
| Postdural puncture headache | 229549 | 129549 | 349 | G97.1 |
| Postdural puncture headaches | 229550 | 129550 | 349 |  |
| Puncture headaches, post-dural | 229551 | 129551 | 349 |  |
| Puncture headaches, post-lumbar | 229552 | 129552 | 349 |  |
| Puncture headaches, postdural | 229553 | 129553 | 349 |  |
| Retro-ocular headache | 229554 | 129554 | 349 |  |
| Headache, ocular | 231826 | 131826 | 784 |  |
| Headache, orthostatic | 231827 | 131827 | 784 |  |
| Headache, periorbital | 231828 | 131828 | 784 |  |
| Headache, post-dural puncture | 231829 | 131829 | 784 |  |
| Headache, post-lumbar puncture | 231830 | 131830 | 784 |  |
| Headache, postdural puncture | 231831 | 131831 | 784 |  |
| Headache, retro-ocular | 231832 | 131832 | 784 | R51.9 |
| Headache, sharp | 231833 | 131833 | 784 |  |
| Postdural puncture headache | 231834 | 131834 | 784 |  |
| Postdural puncture headaches | 231835 | 131835 | 784 |  |
| Puncture headaches, post-dural | 231836 | 131836 | 784 |  |
| Puncture headaches, post-lumbar | 231837 | 131837 | 784 |  |
| Puncture headaches, postdural | 231838 | 131838 | 784 |  |
| Retro-ocular headache | 231839 | 131839 | 784 | R51.9 |
| Pounding headache | 232963 | 132963 | 784 | R51.9 |
| Headache, pounding | 232964 | 132964 | 784 |  |
| Pounding in head | 232965 | 132965 | 784 | R51.9 |
| Cervicogenic migraine | 233600 | 133600 | 346.8 | G43.809 |
| Migraine, cervicogenic | 233601 | 133601 | 346.8 | G43.809 |
| Vascular migraine | 233634 | 133634 | 784 |  |
| Vascular migraine | 233635 | 133635 | 346.9 | G43.909 |
| Intractable classical migraine | 233637 | 133637 | 346.01 | G43.119 |
| Migraine | 233753 | 133753 | 346 |  |
| Migraine | 233755 | 133755 | 346.1 |  |
| Spinal headache complicating labor and delivery | 236539 | 136539 | 668.8 | O74.5 |
| Vertiginous migraine | 236642 | 136642 | 346.80, 780.4 | G43.109 |
| Migraine with vertigo | 236643 | 136643 | 346.80, 780.4 | G43.109 |
| Headache, occipital | 237911 | 137911 | 784 | R51.9 |
| Ocular migraine | 237958 | 137958 | 346.8 | G43.109 |
| Unspecified migraine | 238246 | 138246 | 346.9 |  |
| Vasomotor headache | 243037 | 143037 | 346.9 | G44.009 |
| Headache vasomotor | 243038 | 143038 | 346.9 |  |
| Headaches, post-dural puncture | 245986 | 145986 | 349 |  |
| Headaches, post-dural puncture | 245987 | 145987 | 784 |  |
| Headaches, post-lumbar puncture | 245988 | 145988 | 349 |  |
| Headaches, post-lumbar puncture | 245989 | 145989 | 784 |  |
| Headaches, postdural puncture | 245990 | 145990 | 349 |  |
| Headaches, postdural puncture | 245991 | 145991 | 784 |  |
| Post dural puncture headache | 245992 | 145992 | 349 |  |
| Post-dural puncture headaches | 245993 | 145993 | 349 |  |
| Post-dural puncture headaches | 245994 | 145994 | 784 |  |
| Post-dural puncture headache | 245995 | 145995 | 784 |  |
| Post-lumbar puncture headache | 245996 | 145996 | 349 | G97.1 |
| Post-lumbar puncture headache | 245997 | 145997 | 784 |  |
| Post-lumbar puncture headaches | 245998 | 145998 | 349 |  |
| Post-lumbar puncture headaches | 245999 | 145999 | 784 |  |
| Puncture headache, post-dural | 246000 | 146000 | 349 |  |
| Puncture headache, post-dural | 246001 | 146001 | 784 |  |
| Puncture headache, post-lumbar | 246002 | 146002 | 349 |  |
| Puncture headache, post-lumbar | 246003 | 146003 | 784 |  |
| Puncture headache, postdural | 246004 | 146004 | 349 |  |
| Puncture headache, postdural | 246005 | 146005 | 784 |  |
| Hemicranias | 247042 | 147042 | 346.9 |  |
| Hemicrania migraine | 247043 | 147043 | 346.9 | G43.909 |
| Hemicrania migraines | 247044 | 147044 | 346.9 |  |
| Abdominal migraines | 247045 | 147045 | 346.2 |  |
| Migraines, abdominal | 247046 | 147046 | 346.2 |  |
| Cervical migraine syndrome | 247047 | 147047 | 346 | G43.809 |
| Cervical migraine syndromes | 247048 | 147048 | 346 |  |
| Migraine syndrome, cervical | 247049 | 147049 | 346 |  |
| Migraine syndromes, cervical | 247050 | 147050 | 346 |  |
| Syndrome, cervical migraine | 247051 | 147051 | 346 |  |
| Syndromes, cervical migraine | 247052 | 147052 | 346 |  |
| Variant, migraine | 247063 | 147063 | 346.2 |  |
| Variants, migraine | 247064 | 147064 | 346.2 |  |
| Status migrainosus | 247065 | 147065 | 346.2 | G43.901 |
| Migraine without mention of intractable migraine | 247854 | 147854 | 346.9 | G43.909 |
| Migraine without intractable migraine | 247855 | 147855 | 346.1 | G43.009 |
| Migraine NEC | 248530 | 148530 | 346.8 |  |
| Vidian neuralgia | 250580 | 150580 | 729.2 | G44.009 |
| Chronic daily headache | 255857 | 155857 | 784 | R51.9 |
| Sinus headache | 255985 | 155985 | 784 | R51.9 |
| Muscle contraction headache syndrome | 258890 | 158890 | 307.81 | G44.209 |
| Muscle contraction headache | 258891 | 158891 | 307.81 | G44.209 |
| Trigger point with tension headache | 261084 | 161084 | 307.81 | G44.209 |
| Headaches, sick | 261368 | 161368 | 346.1 |  |
| Sick headaches | 261369 | 161369 | 346.1 |  |
| Ophthalmic migraine | 263023 | 163023 | 346.8 | G43.109 |
| Frontal headache | 264547 | 164547 | 784 | R51.9 |
| Headache disorder | 264548 | 164548 | 784 | R51.9 |
| Occipital headache | 264549 | 164549 | 784 | R51.9 |
| Hemicrania | 265316 | 165316 | 346.9 | G43.909 |
| Abn reaction/later complication lumbar puncture, no procedure mishap | 265451 | 165451 | 349 | G97.1 |
| Migraine equivalent syndrome | 266011 | 166011 | 346 |  |
| Migraine with prolonged aura, intractable | 267102 | 167102 | 346.01 | G43.119 |
| Migraine with prolonged aura, not intractable | 267273 | 167273 | 346 | G43.109 |
| Migraine equivalent | 274659 | 174659 | 346.2 | G43.109 |
| Migraine equivalent syndrome | 274660 | 174660 | 346.2 | G43.109 |
| Spinal puncture headache | 274786 | 174786 | 349 | G97.1 |
| Sleep-related headache | 275926 | 175926 | 784 | R51.9 |
| Intractable ophthalmic migraine | 282699 | 182699 | 346.81 | G43.819 |
| Headache with transient neurologic deficits and CSF lymphocytosis | 282782 | 182782 | 346.8 |  |
| HaNDL (headache, transient neurologic deficits and CSF lymphocytosis) | 282783 | 182783 | 346.8 |  |
| Pseudomigraine | 282784 | 182784 | 346.8 | G43.809 |
| Retrobulbar headache | 283029 | 183029 | 784 | R51.9 |
| Benign exertional headache | 283195 | 183195 | 784 | G44.84 |
| Dental headache | 283295 | 183295 | 784 | R51.9 |
| Aching headache | 283774 | 183774 | 784 | R51.9 |
| Left facial pain | 284353 | 184353 | 784 | R51.9 |
| Right facial pain | 284354 | 184354 | 784 | R51.9 |
| Lt facial pain | 284376 | 184376 | 784 | R51.9 |
| Nocturnal headaches | 284471 | 184471 | 784 | R51.9 |
| Right-sided headache | 284506 | 184506 | 784 | R51.9 |
| Left-sided headache | 284507 | 184507 | 784 | R51.9 |
| Chronic right-sided headaches | 284575 | 184575 | 784 | R51.9, G89.29 |
| Chronic left-sided headaches | 284576 | 184576 | 784 | R51.9, G89.29 |
| New onset headache | 284767 | 184767 | 784 | R51.9 |
| Left-sided face pain | 284776 | 184776 | 784 | R51.9 |
| Right-sided face pain | 284777 | 184777 | 784 | R51.9 |
| Supraorbital headache | 284873 | 184873 | 784 | R51.9 |
| Severe frontal headaches | 285175 | 185175 | 784 | R51.9 |
| Cluster headache syn NOS | 285592 | 185592 | 339 |  |
| Episodc cluster headache | 285593 | 185593 | 339.01 |  |
| Episdc paroxyml hemicran | 285594 | 185594 | 339.03 |  |
| Chr paroxysml hemicrania | 285595 | 185595 | 339.04 |  |
| Trigem autonmc cephl NEC | 285597 | 185597 | 339.09 |  |
| Tension headache NOS | 285598 | 185598 | 307.81 |  |
| Episdic tension headache | 285599 | 185599 | 339.11 |  |
| Chronic tension headache | 285600 | 185600 | 339.12 | G44.229 |
| Drug induce headache NEC | 285604 | 185604 | 339.3 |  |
| Headache w sex activity | 285609 | 185609 | 339.82 |  |
| Prim exertion headache | 285610 | 185610 | 339.84 |  |
| Prim stabbing headache | 285611 | 185611 | 339.85 |  |
| Headache syndrome NEC | 285612 | 185612 | 339.89 |  |
| Headache, stabbing | 286102 | 186102 | 339.85 |  |
| Headaches, stabbing | 286103 | 186103 | 339.85 |  |
| Idiopathic stabbing headache | 286104 | 186104 | 339.85 | G44.85 |
| Stabbing headache | 286105 | 186105 | 339.85 | G44.85 |
| Stabbing headaches | 286106 | 186106 | 339.85 |  |
| Cough headache | 286107 | 186107 | 339.83 | G44.83 |
| Cough headache syndrome | 286108 | 186108 | 339.83 | G44.83 |
| Cough headaches | 286109 | 186109 | 339.83 |  |
| Headache, cough | 286110 | 186110 | 339.83 |  |
| Headaches, cough | 286111 | 186111 | 339.83 |  |
| Chronic tension headaches | 286116 | 186116 | 339.12 | G44.229 |
| Headache, tension | 286117 | 186117 | 307.81 |  |
| Headache, tension-type | 286118 | 186118 | 339.1 | G44.209 |
| Headaches, tension | 286119 | 186119 | 307.81 |  |
| Headaches, tension-type | 286120 | 186120 | 339.1 |  |
| Tension headaches | 286122 | 186122 | 307.81 |  |
| Tension, headache | 286123 | 186123 | 307.81 |  |
| Tension-type headaches | 286124 | 186124 | 339.1 |  |
| Tension type headache, unspecified | 286125 | 186125 | 307.81 |  |
| Tension headache NOS | 286126 | 186126 | 307.81 |  |
| Chronic paroxysmal hemicranias | 286127 | 186127 | 339.04 |  |
| Hemicrania, chronic paroxysmal | 286128 | 186128 | 339.04 |  |
| Hemicranias, chronic paroxysmal | 286129 | 186129 | 339.04 |  |
| Paroxysmal hemicranias, chronic | 286130 | 186130 | 339.04 |  |
| Cluster headaches, episodic | 286131 | 186131 | 339.01 |  |
| Episodic cluster headaches | 286132 | 186132 | 339.01 |  |
| Headache, episodic cluster | 286133 | 186133 | 339.01 |  |
| Headaches, episodic cluster | 286134 | 186134 | 339.01 |  |
| Bing-Horton syndrome | 286135 | 186135 | 339 | G44.009 |
| Cephalalgia, histamine | 286136 | 186136 | 339 |  |
| Cephalalgia, paroxysmal, nocturnal, orbital | 286137 | 186137 | 339 |  |
| Cephalgia, histamine | 286138 | 186138 | 339 |  |
| Cephalgias, histamine | 286139 | 186139 | 339 | G44.009 |
| Cluster headache | 286140 | 186140 | 339 | G44.009 |
| Cluster headache syndrome | 286141 | 186141 | 339 | G44.009 |
| Erythroprosopalgia | 286142 | 186142 | 339 | G44.009 |
| Headache histamine | 286143 | 186143 | 339 |  |
| Headache syndrome, cluster | 286144 | 186144 | 339 |  |
| Headache, cluster | 286145 | 186145 | 339 | G44.009 |
| Headache, histamine | 286146 | 186146 | 339 |  |
| Headaches, cluster | 286147 | 186147 | 339 | G44.009 |
| Histamine cephalgia | 286148 | 186148 | 339 | G44.009 |
| Histamine cephalgias | 286149 | 186149 | 339 | G44.009 |
| Histamine headache | 286150 | 186150 | 339 | G44.009 |
| Horton's headache | 286151 | 186151 | 339 | G44.009 |
| Horton's neuralgia | 286152 | 186152 | 339 | G44.009 |
| Hortons syndrome | 286153 | 186153 | 339 | G44.009 |
| Migraine, neuralgic | 286154 | 186154 | 339 |  |
| Migraines, neuralgic | 286155 | 186155 | 339 | G44.009 |
| Migrainous neuralgia | 286156 | 186156 | 339 | G44.009 |
| Neuralgia, migrainous | 286157 | 186157 | 339 |  |
| Neuralgic migraine | 286158 | 186158 | 339 | G44.009 |
| Neuralgic migraines | 286159 | 186159 | 339 | G44.009 |
| Syndrome, Bing-Horton's | 286160 | 186160 | 339 |  |
| Syndrome, cluster headache | 286161 | 186161 | 339 |  |
| Neuralgia, Sluder | 286369 | 186369 | 337.09 | G44.89 |
| Sluder's neuralgia | 286374 | 186374 | 337.09 | G44.89 |
| Sluder's syndrome | 286375 | 186375 | 337.09 | G44.89 |
| Cluster headaches and other trigeminal autonomic cephalgias | 286389 | 186389 | 339 |  |
| Cluster headaches and other trigeminal autonomic cephalgias | 286390 | 186390 | 339.09 |  |
| Cluster headache/TACS | 286391 | 186391 | 339 |  |
| Cluster headache/TACS | 286392 | 186392 | 339.09 |  |
| TACS (trigeminal autonomic cephalgias) | 286394 | 186394 | 339.09 | G44.099 |
| Ciliary neuralgia | 286395 | 186395 | 339 | G44.009 |
| Ciliary neuralgias | 286396 | 186396 | 339 |  |
| Neuralgia, ciliary | 286397 | 186397 | 339 |  |
| Neuralgias, ciliary | 286398 | 186398 | 339 |  |
| Lower half headache | 286399 | 186399 | 784 | R51.9 |
| Lower half migraine | 286400 | 186400 | 339 | G43.809 |
| Chronic cluster headaches | 286401 | 186401 | 339.02 |  |
| Cluster headaches, chronic | 286402 | 186402 | 339.02 |  |
| Headache, chronic cluster | 286403 | 186403 | 339.02 |  |
| Headaches, chronic cluster | 286404 | 186404 | 339.02 |  |
| Paroxysmal hemicrania | 286405 | 186405 | 339.03 | G44.039 |
| Medication overuse headache | 286406 | 186406 | 339.3 | G44.40 |
| Rebound headache | 286407 | 186407 | 339.3 | G44.40 |
| Analgesic rebound headache | 286408 | 186408 | 339.3, E935.9 | G44.40, T39.95XA |
| Other specified headache syndromes | 286410 | 186410 | 339.89 |  |
| Orgasmic headache | 286411 | 186411 | 339.82 | G44.82 |
| Preorgasmic headache | 286412 | 186412 | 339.82 | G44.82 |
| Basilar artery migraine | 286414 | 186414 | 346 | G43.109 |
| Basilar migraine | 286415 | 186415 | 346 | G43.109 |
| Bickerstaff's migraine | 286416 | 186416 | 346 | G43.109 |
| Migraine, basilar | 286417 | 186417 | 346 |  |
| Vertebro - basilar migraine | 286418 | 186418 | 346 |  |
| Migraine triggered seizures (HCC) | 286419 | 186419 | 346 | G43.109, R56.9 |
| Migraine aura without headache (migraine equivalents) | 286420 | 186420 | 346 | G43.109 |
| Migraine, retinal | 286421 | 186421 | 346 |  |
| Retinal migraine | 286422 | 186422 | 346 | G43.109 |
| Variants of migraine, not elsewhere classified | 286426 | 186426 | 346.2 |  |
| Variants of migraine NEC | 286427 | 186427 | 346.2 |  |
| Cyclical vomiting associated with migraine | 286429 | 186429 | 346.2 | G43.A0 |
| Migraine, ophthalmoplegic | 286430 | 186430 | 346.2 | G43.B0 |
| Ophthalmoplegic migraine | 286431 | 186431 | 346.2 | G43.B0 |
| Ophthalmoplegic migraine headache | 286432 | 186432 | 346.2 | G43.B0 |
| Periodic headache syndromes in child or adolescent | 286434 | 186434 | 346.2 |  |
| Hemiplegic migraine | 286437 | 186437 | 346.3 | G43.409 |
| Familial hemiplegic migraine | 286438 | 186438 | 346.3 | G43.409 |
| Familial hemiplegic migraines | 286439 | 186439 | 346.3 |  |
| Hemiplegic migraine, familial | 286440 | 186440 | 346.3 |  |
| Hemiplegic migraines, familial | 286441 | 186441 | 346.3 |  |
| Migraine, familial hemiplegic | 286442 | 186442 | 346.3 |  |
| Migraines, familial hemiplegic | 286443 | 186443 | 346.3 |  |
| Familial migraine | 286444 | 186444 | 346.3 | G43.409 |
| Sporadic migraine | 286445 | 186445 | 346.3 | G43.409 |
| Menstrual migraine | 286446 | 186446 | 346.4 | G43.829 |
| Migraine - menstrual | 286447 | 186447 | 346.4 |  |
| Menstrual headache | 286448 | 186448 | 346.4 | G43.829 |
| Menstrually related migraine | 286449 | 186449 | 346.4 | G43.829 |
| Pure menstrual migraine | 286450 | 186450 | 346.4 | G43.829 |
| Premenstrual migraine | 286451 | 186451 | 346.4 | G43.829 |
| Premenstrual headache | 286452 | 186452 | 346.4 | G43.829 |
| Persistent migraine aura without cerebral infarction | 286453 | 186453 | 346.5 | G43.509 |
| Persistent migraine aura | 286454 | 186454 | 346.5 | G43.509 |
| Persistent migraine aura with cerebral infarction (HCC) | 286456 | 186456 | 346.60, 434.91 | G43.609, I63.9 |
| Pers migraine aura/cerebral infarct | 286457 | 186457 | 346.6 |  |
| Chronic migraine without aura | 286459 | 186459 | 346.7 | G43.709 |
| Transformed migraine without aura | 286460 | 186460 | 346.7 | G43.709 |
| Allergic migraine | 287159 | 187159 | 346.2 | G43.809 |
| Headache associated with sexual activity type 2 | 287285 | 187285 | 339.82 | G44.82 |
| Headache associated with orgasm | 287286 | 187286 | 339.82 | G44.82 |
| Headache associated with sexual activity type 1 | 287288 | 187288 | 339.82 | G44.82 |
| Pre-orgasmic headache | 287290 | 187290 | 339.82 | G44.82 |
| Exertional headache, primary | 287349 | 187349 | 339.84 | G44.84 |
| Mobius' disease (ophthalmoplegic migraine) | 290024 | 190024 | 346.2 | G43.B0 |
| Moebius' disease (ophthalmoplegic migraine) | 290025 | 190025 | 346.2 | G43.B0 |
| Allergic headache | 290040 | 190040 | 339 | G44.89 |
| Headache, allergic | 290041 | 190041 | 339 |  |
| Headache, sick | 290042 | 190042 | 346.9 |  |
| Headaches, sick | 290043 | 190043 | 346.9 |  |
| Sick headache | 290044 | 190044 | 346.9 | G43.909 |
| Sick headaches | 290045 | 190045 | 346.9 |  |
| Benign headache | 290046 | 190046 | 784 | R51.9 |
| Headache, tension-vascular | 290047 | 190047 | 339.1 |  |
| Headaches, tension-vascular | 290048 | 190048 | 339.1 |  |
| Muscle tension headache | 290049 | 190049 | 339.1 |  |
| Tension vascular headache | 290050 | 190050 | 339.1 |  |
| Tension-type headache | 290051 | 190051 | 339.1 | G44.209 |
| Tension-vascular headache | 290052 | 190052 | 339.1 |  |
| Tension-vascular headaches | 290053 | 190053 | 339.1 |  |
| Atypical migraine | 290109 | 190109 | 346.8 | G43.009 |
| Migraine, atypical | 290110 | 190110 | 346.8 |  |
| Headache | 290335 | 190335 | 339.03 |  |
| Headache | 290337 | 190337 | 339.04 |  |
| Headache | 290338 | 190338 | 339.05 |  |
| Headache | 290343 | 190343 | 339.09 |  |
| Muscle tension headache | 290356 | 190356 | 339.11 |  |
| Muscle tension headache | 290357 | 190357 | 339.12 |  |
| Drug induced headache | 290367 | 190367 | 339.3, E980.5 | G44.40 |
| Headache | 290389 | 190389 | 339.81 |  |
| Headache | 290390 | 190390 | 339.89 |  |
| Headache syndromes | 290391 | 190391 | 339.89 |  |
| Intractable migraine with aura | 290393 | 190393 | 346.01 | G43.119 |
| Intractable migraine with aura with status migrainosus | 290397 | 190397 | 346.03 | G43.111 |
| Intractable migraine without aura | 290398 | 190398 | 346.11 | G43.019 |
| Intractable migraine without aura with status migrainosus | 290400 | 190400 | 346.13 | G43.011 |
| Variants of migraine with status migrainosus | 290403 | 190403 | 346.22 | G43.801 |
| Intractable hemiplegic migraine | 290406 | 190406 | 346.31 | G43.419 |
| Hemiplegic migraine with status migrainosus | 290407 | 190407 | 346.32 | G43.401 |
| Intractable hemiplegic migraine with status migrainosus | 290409 | 190409 | 346.33 | G43.411 |
| Intractable menstrual migraine | 290411 | 190411 | 346.41 | G43.839 |
| Menstrual migraine with status migrainosus | 290413 | 190413 | 346.42 | G43.821 |
| Intractable menstrual migraine with status migrainosus | 290415 | 190415 | 346.43 | G43.831 |
| Intractable persistent migraine aura | 290417 | 190417 | 346.51 | G43.519 |
| Persistent migraine aura with status migrainosus | 290420 | 190420 | 346.52 | G43.501 |
| Intractable persistent migraine aura with status migrainosus | 290422 | 190422 | 346.53 | G43.511 |
| Short unilateral neuralgiform headache, conjunctival injection/tearing | 290482 | 190482 | 339.05 | G44.059 |
| Persistent migraine aura with stroke (HCC) | 290484 | 190484 | 346.60, 434.91 | G43.609, I63.9 |
| Intractable persistent migraine aura with cerebral infarction (HCC) | 290486 | 190486 | 346.61, 434.91 | G43.619, I63.9 |
| Persistent migraine aura with cerebral infarction, status migrainosus (HCC) | 290489 | 190489 | 346.62, 434.91 | G43.601, I63.9 |
| Intractable chronic migraine without aura | 290492 | 190492 | 346.71 | G43.719 |
| Chronic migraine without aura, with status migrainosus | 290494 | 190494 | 346.72 | G43.701 |
| Intractable chronic migraine without aura with status migrainosus | 290496 | 190496 | 346.73 | G43.711 |
| Intractable migraine with status migrainosus | 290510 | 190510 | 346.93 | G43.911 |
| Persistent migraine aura, cerebral infarction, intractable, in status (HCC) | 290573 | 190573 | 346.63, 434.91 | G43.611, I63.9 |
| Trigeminal autonomic cephalgias | 291012 | 191012 | 339.09 | G44.099 |
| Primary headache associated with sexual activity | 291688 | 191688 | 339.82 | G44.82 |
| TTH (tension-type headache) | 291689 | 191689 | 339.1 | G44.209 |
| Chronic tension-type headache, not intractable | 291690 | 191690 | 339.12 | G44.229 |
| Chronic tension-type headache, intractable | 291691 | 191691 | 339.12 | G44.221 |
| Vertebro-basilar migraine | 292054 | 192054 | 346 | G43.109 |
| Vertebrobasilar migraine | 292055 | 192055 | 346 | G43.109 |
| Drug-induced headache, not elsewhere classified | 292996 | 192996 | 339.3 |  |
| Other migraine | 294052 | 194052 | 346.8 |  |
| Migraine with aura (classical migraine) | 294329 | 194329 | 346 |  |
| Migraine without aura (common migraine) | 294330 | 194330 | 346.1 |  |
| Migraine unspecified | 297991 | 197991 | 346.9 |  |
| Drug-induced headache nec | 298401 | 198401 | 339.3 |  |
| Basilar artery migraine, intractable | 300209 | 200209 | 346.01 | G43.119 |
| Intractable basilar artery migraine | 300210 | 200210 | 346.01 | G43.119 |
| Headache with transient neurologic deficits and CSF lymphocytosis | 300570 | 200570 | 784.0, 288.61, 435.9 | R51.9, D72.820, R29.818 |
| HaNDL (headache, transient neurologic deficits and CSF lymphocytosis) | 300571 | 200571 | 784.0, 288.61, 435.9 | R51.9, D72.820, R29.818 |
| Intractable episodic tension-type headache | 300686 | 200686 | 339.11 | G44.211 |
| Episodic tension-type headache, intractable | 300687 | 200687 | 339.11 | G44.211 |
| Headache, vasomotor | 300932 | 200932 | 346.9 | G44.009 |
| Frequent episodic tension-type headache | 303489 | 203489 | 339.11 | G44.219 |
| Headache, frequent episodic tension-type | 303490 | 203490 | 339.11 | G44.219 |
| TTH (tension-type headache), frequent episodic type | 303491 | 203491 | 339.11 | G44.219 |
| Frequent episodic tension-type headache, not intractable | 303492 | 203492 | 339.11 | G44.219 |
| Infrequent episodic tension-type headache | 303538 | 203538 | 339.11 | G44.219 |
| Headache, infrequent episodic tension-type | 303539 | 203539 | 339.11 | G44.219 |
| TTH (tension-type headache), infrequent episodic type | 303540 | 203540 | 339.11 | G44.219 |
| Infrequent episodic tension-type headache, not intractable | 303541 | 203541 | 339.11 | G44.219 |
| Chronic facial pain | 303636 | 203636 | 784.0, 338.29 | R51.9, G89.29 |
| Chronic pain in face | 303637 | 203637 | 784.0, 338.29 | R51.9, G89.29 |
| Chronic face pain | 303638 | 203638 | 784.0, 338.29 | R51.9, G89.29 |
| One-sided headache | 304815 | 204815 | 784 | R51.9 |
| Emotional tension headache | 305295 | 205295 | 307.81 | G44.209 |
| Headache, emotional tension | 305296 | 205296 | 307.81 | G44.209 |
| Chronic mixed headache syndrome | 306292 | 206292 | 339.89 | G44.89 |
| Cluster headache, not intractable | 306588 | 206588 | 339 | G44.009 |
| Anterior ethmoidal nerve syndrome | 306618 | 206618 | 337.09 | G44.89 |
| Anterior ethmoidal syndrome | 306619 | 206619 | 337.09 | G44.89 |
| Hemiplegic migraine, intractable | 306929 | 206929 | 346.31 | G43.419 |
| Headache with watery eyes | 307746 | 207746 | 339.05 |  |
| Atypical cluster headache | 308043 | 208043 | 339 | G44.009 |
| Atypical cluster headaches | 308044 | 208044 | 339 |  |
| Cluster headache, atypical | 308045 | 208045 | 339 |  |
| Cluster headaches, atypical | 308046 | 208046 | 339 |  |
| Headache, atypical cluster | 308047 | 208047 | 339 |  |
| Headaches, atypical cluster | 308048 | 208048 | 339 |  |
| Intractable cluster headache | 308049 | 208049 | 339 | G44.001 |
| Cluster headache, intractable | 308050 | 208050 | 339 | G44.001 |
| Spinal headache complicating labor and delivery, delivered | 308609 | 208609 | 668.81, 349.0 | O74.5 |
| Spinal headache complicating labor and delivery, delivered, postpartum | 308610 | 208610 | 668.84 | O74.5 |
| Spinal headache complicating labor and delivery, antepartum | 308611 | 208611 | 668.83, 349.0 | O74.5 |
| Spinal headache complicating labor and delivery, postpartum condition | 308612 | 208612 | 668.82, 349.0 | O74.5 |
| Headache behind the eyes | 308746 | 208746 | 784 | R51.9 |
| Headache around the eyes | 308901 | 208901 | 784 | R51.9 |
| Postoperative spinal headache | 309336 | 209336 | 349 | G97.1 |
| Headaches at night | 309584 | 209584 | 784 |  |
| Headache, cluster, chronic | 309634 | 209634 | 339.02 | G44.029 |
| Headache, paroxysmal hemicrania, chronic | 309637 | 209637 | 339.04 | G44.049 |
| Headache, cluster, episodic | 309677 | 209677 | 339.01 | G44.019 |
| Headache, hemiplegic migraine | 309691 | 209691 | 346.3 | G43.409 |
| Headache, menstrual migraine | 309708 | 209708 | 346.4 | G43.829 |
| Headache, variant migraine | 309710 | 209710 | 346.2 | G43.809 |
| Headache, variant migraine, intractable | 309855 | 209855 | 346.21 | G43.819 |
| Headache, tension type, chronic | 309921 | 209921 | 339.12 | G44.229 |
| Headache, migraine, with status migrainosus | 309975 | 209975 | 346.92 | G43.901 |
| Headache, hemiplegic migraine, intractable, with status migrainosus | 309977 | 209977 | 346.33 | G43.411 |
| Headache, hemiplegic migraine, intractable | 309978 | 209978 | 346.31 | G43.419 |
| Headache, variant migraine, with status migrainosus | 309980 | 209980 | 346.22 | G43.801 |
| Headache, tension type, episodic | 309984 | 209984 | 339.11 | G44.219 |
| Headache, paroxysmal hemicrania, episodic | 309985 | 209985 | 339.03 | G44.039 |
| Headache, drug induced | 309999 | 209999 | 339.3, E980.5 | G44.40 |
| Headache, spinal, postoperative | 310015 | 210015 | 349 | G97.1 |
| Headache, cluster, intractable | 310291 | 210291 | 339 | G44.001 |
| Headache, classical migraine, intractable | 310314 | 210314 | 346.01 | G43.119 |
| Headache, common migraine, intractable | 310315 | 210315 | 346.11 | G43.019 |
| Headache, hemiplegic migraine, with status migrainosus | 313487 | 213487 | 346.32 | G43.401 |
| Spinal tap complication | 313579 | 213579 | 349 |  |
| Headache, migraine, intractable, with status migrainosus | 317480 | 217480 | 346.93 | G43.911 |
| Headache, chronic migraine without aura, intractable, with status | 317481 | 217481 | 346.73 | G43.711 |
| Headache, chronic migraine without aura, with status migrainosus | 317482 | 217482 | 346.72 | G43.701 |
| Headache, chronic migraine without aura, intractable | 317483 | 217483 | 346.71 | G43.719 |
| Persistent migraine aura with cerebral infarct, intractable (HCC) | 317484 | 217484 | 346.61, 434.91 | G43.619, I63.9 |
| Persistent migraine aura, intractable, with status migrainosus | 317485 | 217485 | 346.53 | G43.511 |
| Persistent migraine aura, intractable | 317486 | 217486 | 346.51 | G43.519 |
| Headache, menstrual migraine, intractable, with status migrainosus | 317487 | 217487 | 346.43 | G43.831 |
| Headache, menstrual migraine, with status migrainosus | 317488 | 217488 | 346.42 | G43.821 |
| Headache, menstrual migraine, intractable | 317489 | 217489 | 346.41 | G43.839 |
| Headache, common migraine, intractable, with status migrainosus | 317490 | 217490 | 346.13 | G43.011 |
| Headache, common migraine, with status migrainosus | 317491 | 217491 | 346.12 | G43.001 |
| Headache, classical migraine, with status migrainosus | 317493 | 217493 | 346.02 | G43.101 |
| Headache, chronic migraine without aura | 318833 | 218833 | 346.7 | G43.709 |
| Headache , short unilat neuralgiform, w/conjunctival injection/tearing | 318834 | 218834 | 339.05 | G44.059 |
| Headache, classical migraine | 318835 | 218835 | 346 | G43.109 |
| Headache, common migraine | 318836 | 218836 | 346.1 | G43.009 |
| Headache, trigeminal autonomic | 318838 | 218838 | 339.09 | G44.099 |
| Refractory migraine without aura | 323682 | 223682 | 346.11 | G43.019 |
| Postpartum spinal headache | 323969 | 223969 | 668.84 | O89.4 |
| Cervicogenic headache | 323985 | 223985 | 784 | G44.86 |
| Chronic headache disorder | 324020 | 224020 | 784 | R51.9, G89.29 |
| Pregnancy headache, postpartum | 333345 | 233345 | 646.84, 784.0 | O90.89, R51.9 |
| Headache in pregnancy, postpartum | 333346 | 233346 | 646.84, 784.0 | O90.89, R51.9 |
| Pregnancy headache, postpartum | 333347 | 233347 | 784 |  |
| Headache in pregnancy, postpartum | 333348 | 233348 | 784 |  |
| Pregnancy headache, delivered with postpartum complication | 333349 | 233349 | 784 |  |
| Pregnancy headache, delivered with postpartum complication | 333350 | 233350 | 646.82, 784.0 | O26.899, R51.9 |
| Headache in pregnancy, delivered with postpartum complication | 333351 | 233351 | 646.82, 784.0 | O26.899, R51.9 |
| Headache in pregnancy, delivered with postpartum complication | 333352 | 233352 | 784 |  |
| Pregnancy headache, antepartum | 333353 | 233353 | 784 |  |
| Pregnancy headache, antepartum | 333354 | 233354 | 646.83, 784.0 | O26.899, R51.9 |
| Headache in pregnancy, antepartum | 333355 | 233355 | 646.83, 784.0 | O26.899, R51.9 |
| Headache in pregnancy, antepartum | 333356 | 233356 | 784 |  |
| Pregnancy headache | 333357 | 233357 | 784 |  |
| Headache in pregnancy | 333358 | 233358 | 784 |  |
| Pregnancy headache | 333359 | 233359 | 646.80, 784.0 | O26.899, R51.9 |
| Headache in pregnancy | 333360 | 233360 | 646.80, 784.0 | O26.899, R51.9 |
| Chronic headaches | 335439 | 235439 | 784 | R51.9, G89.29 |
| Headache, lumbar puncture | 336080 | 236080 | 349 | G97.1 |
| Head and face pain | 337932 | 237932 | 784 | R51.9 |
| Pain, head and face | 337933 | 237933 | 784 | R51.9 |
| Headache, post-myelogram | 346720 | 246720 | 349 | G97.1 |
| Migraine headache without aura | 349893 | 249893 | 346.1 | G43.009 |
| Classic migraine with aura | 350101 | 250101 | 346 | G43.109 |
| Common migraine without aura | 350103 | 250103 | 346.1 | G43.009 |
| Daily headache | 350108 | 250108 | 784 | R51.9 |
| New onset of headaches | 350150 | 250150 | 784 | R51.9 |
| New onset of headaches after age 50 | 350299 | 250299 | 784 | R51.9 |
| Sudden onset of severe headache | 350340 | 250340 | 784 | R51.9 |
| Headache causing frequent awakening from sleep | 350418 | 250418 | 784 | R51.9 |
| Headache upon awakening | 350419 | 250419 | 784 | R51.9 |
| Increased frequency of headaches | 350427 | 250427 | 784 | R51.9 |
| Increased severity of headaches | 350428 | 250428 | 784 | R51.9 |
| New onset of headache in cancer patient | 350447 | 250447 | 784.0, 199.1 | R51.9 |
| New onset of headache in immunocompromised patient (HCC) | 350448 | 250448 | 784.0, 279.9 | R51.9, D84.9 |
| Persistent headaches | 350449 | 250449 | 784 | R51.9 |
| Worsening headaches | 350465 | 250465 | 784 | R51.9 |
| Intractable chronic migraine without aura and with status migrainosus | 350594 | 250594 | 346.73 | G43.711 |
| Intractable migraine with aura without status migrainosus | 350595 | 250595 | 346.01 | G43.119 |
| Intractable migraine without aura and with status migrainosus | 350597 | 250597 | 346.13 | G43.011 |
| Other headache syndrome | 350670 | 250670 | 339.89 |  |
| Analgesic overuse headache | 351698 | 251698 | 339.3, E935.9 | G44.40, T39.95XA |
| Headache, acute | 351939 | 251939 | 784 | R51.9 |
| Acute headache | 351940 | 251940 | 784 | R51.9 |
| Common transformed migraine without aura | 352623 | 252623 | 346.1 | G43.709 |
| Exertional headache | 353169 | 253169 | 784 | G44.84 |
| Migraine with visual aura | 354134 | 254134 | 346 | G43.109 |
| Mixed headache | 354149 | 254149 | 784 | R51.9 |
| Non-ocular headache | 354503 | 254503 | 784 | R51.9 |
| Occipital pain | 354642 | 254642 | 784 | R51.9 |
| Pain of occiput | 354643 | 254643 | 784 | R51.9 |
| Pain of postauricular region | 354648 | 254648 | 784 | R51.9 |
| Postauricular pain | 354649 | 254649 | 784 | R51.9 |
| Positional headache | 354807 | 254807 | 784 | R51.0 |
| Status migrainosus without intractable migraine | 355340 | 255340 | 346.92 | G43.901 |
| Trigger point of head | 355779 | 255779 | 784 | R51.9 |
| Morning headache | 357855 | 257855 | 784 | R51.9 |
| Pain of cheek | 358014 | 258014 | 784 | R51.9 |
| Pain of scalp | 358028 | 258028 | 784 | R51.9 |
| Scalp pain | 358029 | 258029 | 784 | R51.9 |
| Rhinogenic headache | 358237 | 258237 | 784 | R51.9 |
| Scalp tenderness | 358260 | 258260 | 784 | R51.9 |
| Temporal pain | 358521 | 258521 | 784 | R51.9 |
| Tenderness of temple region | 358532 | 258532 | 784 | R51.9 |
| Temple tenderness | 358533 | 258533 | 784 | R51.9 |
| Spinal and epidural anesthesia induced headache during pregnancy | 359382 | 259382 | 668.8 | O29.40 |
| Worst headache of life | 359407 | 259407 | 784 | R51.9 |
| Headache in back of head | 359420 | 259420 | 784 | R51.9 |
| Headache in front of head | 359421 | 259421 | 784 | R51.9 |
| Headache above the eye region | 359422 | 259422 | 784 | R51.9 |
| Headache on top of head | 359423 | 259423 | 784 | R51.9 |
| Orofacial pain | 360812 | 260812 | 528.9, 784.0 | K13.79, R51.9 |
| Headache, classical migraine, intractable, with status migrainosus | 362377 | 262377 | 346.03 | G43.111 |
| Migraine aura, persistent, with cerebral infarct, status migrainosus (HCC) | 362549 | 262549 | 346.62, 434.91 | G43.601, I63.9 |
| Migraine aura, persistent, with cerebral infarct, intractable, status (HCC) | 362550 | 262550 | 346.63, 434.91 | G43.611, I63.9 |
| Post-procedural headache | 362759 | 262759 | 998.89, 784.0 | T81.89XA, R51.9 |
| Post-procedural headache | 362760 | 262760 | 784 |  |
| Headache following intrapartum spinal anesthesia | 363216 | 263216 | 668.80, 349.0 | O29.40 |
| Blinding headache | 363370 | 263370 | 784 | R51.9 |
| Pressure in head | 363786 | 263786 | 784 | R51.9 |
| Tension headache, chronic | 365285 | 265285 | 339.12 | G44.229 |
| Cluster headache syndrome, not intractable | 367214 | 267214 | 339 | G44.009 |
| Chronic cluster headache, not intractable | 368112 | 268112 | 339.02 | G44.029 |
| Chronic paroxysmal hemicrania, not intractable | 368126 | 268126 | 339.04 | G44.049 |
| Drug-induced headache, not elsewhere classified, not intractable | 368378 | 268378 | 339.3 |  |
| Episodic cluster headache, not intractable | 368474 | 268474 | 339.01 | G44.019 |
| Episodic paroxysmal hemicrania, not intractable | 368475 | 268475 | 339.03 | G44.039 |
| Episodic tension-type headache, not intractable | 368476 | 268476 | 339.11 | G44.219 |
| Intractable vascular headache | 371876 | 271876 | 784 | G44.1 |
| Other intractable trigeminal autonomic cephalgias (TAC) | 372760 | 272760 | 339.09 |  |
| Intractable hemiplegic migraine without status migrainosus | 373263 | 273263 | 346.31 | G43.419 |
| Intractable chronic paroxysmal hemicrania | 373941 | 273941 | 339.04 | G44.041 |
| Migraine with status migrainosus, not intractable | 373979 | 273979 | 346.92 | G43.901 |
| Ophthalmoplegic migraine without status migrainosus, not intractable | 374073 | 274073 | 346.2 | G43.B0 |
| Tension-type headache, not intractable | 375016 | 275016 | 339.1 | G44.209 |
| Migraine with aura and with status migrainosus, not intractable | 375375 | 275375 | 346.03 | G43.101 |
| Other trigeminal autonomic cephalgia (TAC), not intractable | 375574 | 275574 | 339.09 |  |
| Intractable cyclical vomiting with status migrainosus | 376022 | 276022 | 346.23 | G43.A1, G43.901 |
| Intractable cluster headache syndrome | 377388 | 277388 | 339 | G44.001 |
| Intractable drug-induced headache, not elsewhere classified | 378019 | 278019 | 339.3 |  |
| Migraine without status migrainosus, not intractable | 380205 | 280205 | 346.9 | G43.909 |
| Hemiplegic migraine with status migrainosus, not intractable | 383489 | 283489 | 346.32 | G43.401 |
| Cyclical vomiting without status migrainosus, not intractable | 384114 | 284114 | 346.2 | G43.A0 |
| Menstrual migraine without status migrainosus, not intractable | 384293 | 284293 | 346.4 | G43.829 |
| Hemiplegic migraine without status migrainosus, not intractable | 385610 | 285610 | 346.3 | G43.409 |
| Migraine without aura and without status migrainosus, not intractable | 385663 | 285663 | 346.1 | G43.009 |
| Vascular headache, not intractable | 386736 | 286736 | 784 | G44.1 |
| Other migraine without status migrainosus, not intractable | 387194 | 287194 | 346.8 |  |
| Menstrual migraine with status migrainosus, not intractable | 387733 | 287733 | 346.42 | G43.821 |
| Intractable menstrual migraine without status migrainosus | 388440 | 288440 | 346.41 | G43.839 |
| Intractable migraine without aura and without status migrainosus | 389169 | 289169 | 346.11 | G43.019 |
| Migraine with aura and without status migrainosus, not intractable | 389916 | 289916 | 346 | G43.109 |
| Intractable episodic cluster headache | 392005 | 292005 | 339.01 | G44.011 |
| Intractable episodic paroxysmal hemicrania | 392006 | 292006 | 339.03 | G44.031 |
| Migraine without aura and with status migrainosus, not intractable | 392040 | 292040 | 346.12 | G43.001 |
| Intractable chronic cluster headache | 392653 | 292653 | 339.02 | G44.021 |
| Intractable tension-type headache | 392655 | 292655 | 339.1 | G44.201 |
| Chronic migraine without aura with status migrainosus, not intractable | 393189 | 293189 | 346.72 | G43.701 |
| Post-seizure headache | 395945 | 295945 | 339.89 | G44.89 |
| Depression headache | 396441 | 296441 | 339.89, 311 | G44.89, F32.A |
| Food sensitivity headache | 396681 | 296681 | 784 | G44.89 |
| Migraine w aura | 399335 | 299335 | 346 |  |
| Migraine wo aura | 399336 | 299336 | 346.1 |  |
| Tension headache, chronic type | 399447 | 299447 | 339.12 |  |
| Migraine, transformed | 399465 | 299465 | 346.7 | G43.709 |
| Headache, cervicogenic | 399497 | 299497 | 784 | G44.86 |
| Headache, chronic daily | 399559 | 299559 | 784 | R51.9 |
| Cluster headache syndrome, intractable | 402332 | 302332 | 339 | G44.001 |
| Other reaction to spinal and lumbar puncture | 410998 | 310998 | 349 |  |
| Oth reaction to spinal & lumbar puncture | 410999 | 310999 | 349 |  |
| Spinal and epidural anesthesia-induced headache during puerperium | 412111 | 312111 | 668.84, 349.0 | O89.4 |
| Complicated migraine | 414434 | 314434 | 346 | G43.109 |
| Complicated migraines | 414435 | 314435 | 346 |  |
| Migraine, complicated | 414436 | 314436 | 346 |  |
| Migraines, complicated | 414437 | 314437 | 346 |  |
| Allergic migraine with status migrainosus | 416281 | 316281 | 346.22 | G43.801 |
| Right sided facial pain | 416576 | 316576 | 784 | R51.9 |
| Intractable common migraine | 416963 | 316963 | 346.11 | G43.019 |
| Complication of lumbar puncture | 418661 | 318661 | 349 | G97.1 |
| Moderate headache | 425535 | 325535 | 784 | R51.9 |
| Mild headache | 425630 | 325630 | 784 | R51.9 |
| Intractable headache | 425656 | 325656 | 784 | R51.9 |
| Abdominal migraine, not intractable | 425832 | 325832 | 346.2 | G43.D0 |
| Postictal headache | 425865 | 325865 | 339.89 | G44.89 |
| Periodic headache syndromes in child or adult, not intractable | 426227 | 326227 | 346.2 |  |
| Ophthalmoplegic migraine, not intractable | 426357 | 326357 | 346.2 | G43.B0 |
| Left temporal headache | 426387 | 326387 | 784 | R51.9 |
| Intractable periodic headache syndrome | 427259 | 327259 | 346.21 | G43.C1 |
| Periodic headache syndrome | 427260 | 327260 | 346.2 | G43.C0 |
| Periodic headache syndrome, not intractable | 427261 | 327261 | 346.2 | G43.C0 |
| Intractable periodic headache syndrome with status migrainosus | 427262 | 327262 | 346.23 | G43.C1 |
| Intractable periodic headache syndrome without status migrainosus | 427263 | 327263 | 346.21 | G43.C1 |
| Periodic headache syndrome with status migrainosus, not intractable | 427264 | 327264 | 346.22 | G43.C0 |
| Periodic headache syndrome without status migrainosus, not intractable | 427265 | 327265 | 346.2 | G43.C0 |
| Other vascular headache | 427266 | 327266 | 784 |  |
| Vascular headache, not elsewhere classified | 427267 | 327267 | 784 |  |
| Migraine headache with aura | 428116 | 328116 | 346 | G43.109 |
| Right temporal headache | 429950 | 329950 | 784 | R51.9 |
| Right sided temporal headache | 429951 | 329951 | 784 | R51.9 |
| Left facial pressure and pain | 430401 | 330401 | 784 | R51.9 |
| Pressure and pain of left side of face | 430402 | 330402 | 784 | R51.9 |
| Migraine NEC/not intrcbl | 436380 | 336380 | 346.8 |  |
| Lumbar puncture as the cause of abnormal reaction of patient, or of later complication, without misadventure at the time of procedure | 438632 | 338632 | 349 | G97.1 |
| Migraine preceded or accompanied by transient focal neurological phenomena | 439687 | 339687 | 346 |  |
| Spinal and epidural anesthesia-induced headache during labor and delivery | 440337 | 340337 | 668.8 | O74.5 |
| Migraine without mention of intractable migraine with status migrainosus | 440960 | 340960 | 346.92 | G43.901 |
| SUNCT (short lasting unilateral neuralgiform headache with conjunctival injection and tearing) | 440972 | 340972 | 339.05 |  |
| Vrnt mgrn wo ntr mgr NEC | 440974 | 340974 | 346.2 |  |
| Intractable chronic migraine without aura and without status migrainosus | 443695 | 343695 | 346.71 | G43.719 |
| Persistent migraine aura without cerebral infarction with intractable migraine | 444171 | 344171 | 346.51 | G43.519 |
| Intractable persistent migraine aura with cerebral infarction and status migrainosus (HCC) | 444390 | 344390 | 346.63, 434.91 | G43.611, I63.9 |
| Intractable persistent migraine aura without cerebral infarction and with status migrainosus | 444391 | 344391 | 346.53 | G43.511 |
| Intractable persistent migraine aura without cerebral infarction and without status migrainosus | 444392 | 344392 | 346.51 | G43.519 |
| Intractable short lasting unilateral neuralgiform headache with conjunctival injection and tearing (SUNCT) | 444393 | 344393 | 339.05 | G44.051 |
| Short lasting unilateral neuralgiform headache with conjunctival injection and tearing (SUNCT), not intractable | 444797 | 344797 | 339.05 | G44.059 |
| Persistent migraine aura with cerebral infarction and without status migrainosus, not intractable (HCC) | 448241 | 348241 | 346.60, 434.91 | G43.609, I63.9 |
| Intractable persistent migraine aura with cerebral infarction and without status migrainosus (HCC) | 449133 | 349133 | 346.61, 434.91 | G43.619, I63.9 |
| Persistent migraine aura with cerebral infarction and status migrainosus, not intractable (HCC) | 450051 | 350051 | 346.62, 434.91 | G43.601, I63.9 |
| Spinal and epidural anesthesia induced headache during pregnancy in first trimester | 450457 | 350457 | 646.83 | O29.41 |
| Spinal and epidural anesthesia induced headache during pregnancy in third trimester | 451148 | 351148 | 646.83 | O29.43 |
| Chronic migraine without aura without status migrainosus, not intractable | 451205 | 351205 | 346.7 | G43.709 |
| Spinal and epidural anesthesia induced headache during pregnancy in second trimester | 452214 | 352214 | 646.83 | O29.42 |
| Persistent migraine aura without cerebral infarction and without status migrainosus, not intractable | 453655 | 353655 | 346.5 | G43.509 |
| Persistent migraine aura without cerebral infarction and with status migrainosus, not intractable | 454773 | 354773 | 346.52 | G43.501 |
| Food sensitivity headache | 456974 | 356974 | 339 |  |
| Chronic head pain | 461217 | 361217 | 784.0, 338.29 | R51.9, G89.29 |
| Head pain, chronic | 461218 | 361218 | 784.0, 338.29 | R51.9, G89.29 |
| Coital headache | 461225 | 361225 | 339.82 | G44.82 |
| Acute facial pain | 461368 | 361368 | 784 | R51.9 |
| Facial pain, acute | 461369 | 361369 | 784 | R51.9 |
| Headache, rebound | 464555 | 364555 | 339.3 | G44.40 |
| Headache, worsening | 464556 | 364556 | 784 | R51.9 |
| Headache, temporal | 465109 | 365109 | 784 | R51.9 |
| Temporal headache | 465110 | 365110 | 784 | R51.9 |
| Headache associated with hormonal factors | 466395 | 366395 | 339.89 | G44.89 |
| Chronic migraine w/o aura w/o status migrainosus, not intractable | 469376 | 369376 | 346.7 | G43.709 |
| Mixed migraine and muscle contraction headache | 472195 | 372195 | 346.90, 307.81 | G43.909, G44.209 |
| Recurrent headache | 472592 | 372592 | 784 | R51.9 |
| Mixed migraine and muscle contraction headache | 472805 | 372805 | 307.81 |  |
| Cervicogenic migraine with intractable migraine and without status migrainosus | 473373 | 373373 | 346.81 | G43.819 |
| Spinal and epidural anaesthesia-induced headache during pregnancy | 476021 | 376021 | 349 |  |
| Spinal and epidural anesthesia-induced headache during labor and delivery | 476022 | 376022 | 349 |  |
| Spinal headache complicating labor and delivery | 476023 | 376023 | 349 |  |
| Spinal and epidural anesthesia induced headache during pregnancy | 476024 | 376024 | 349 |  |
| Spinal and epidural anaesthesia-induced headache during labour and delivery | 476026 | 376026 | 349 |  |
| Periodic headache syndrome in child or adult without status migrainosus, not intractable | 478957 | 378957 | 346.2 |  |
| Morning headache, controlled | 479237 | 379237 | 784 | R51.9 |
| Uncontrolled morning headache | 479249 | 379249 | 784 | R51.9 |
| Unilateral occipital headache | 479263 | 379263 | 784 | R51.9 |
| Migraine NOS/not intrcbl | 480822 | 380822 | 346.9 |  |
| Shrt lst uni nral hdache | 482346 | 382346 | 339.05 |  |
| Hmplg mgr wo ntrc wo st | 482347 | 382347 | 346.3 |  |
| Menst mgr wo ntrc wo st | 482348 | 382348 | 346.4 |  |
| Prst aura wo inf/ntr/st | 482350 | 382350 | 346.5 |  |
| Prs ara w inf wo ntr/st | 482351 | 382351 | 346.6 |  |
| Ch mgr wo ar wo nt wo st | 482352 | 382352 | 346.7 |  |
| Mgrn w aura wo ntrc mgrn | 482387 | 382387 | 346 |  |
| Mgrn wo aura wo intrc mgr | 482388 | 382388 | 346.1 |  |
| Forehead pain | 483053 | 383053 | 784 | R51.9 |
| Migrainous headache without aura | 484837 | 384837 | 346.1 | G43.009 |
| Headache with transient neurologic deficits and cerebrospinal fluid lymphocytosis | 485603 | 385603 | 784.0, 435.9, 288.61 | R51.9, D72.820, R29.818 |
| Intractable allergic migraine | 486762 | 386762 | 346.21 | G43.819 |
| Intractable retinal migraine | 486763 | 386763 | 346.01 | G43.119 |
| Generalised headache | 489775 | 389775 | 784 |  |
| Generalised headaches | 489776 | 389776 | 784 |  |
| Spinal and epidural anaesthesia induced headache during pregnancy | 491824 | 391824 | 349 |  |
| Spinal headache complicating labour and delivery | 491826 | 391826 | 349 |  |
| Intractable migraine with visual aura and without status migrainosus | 505404 | 405404 | 346.01 | G43.119 |
| Migraine variants, not intractable | 505629 | 405629 | 346.2 | G43.809 |
| Migraine without aura, not refractory | 505698 | 405698 | 346.1 | G43.009 |
| Headache Due To Intracranial Disease | 512151 | 412151 | 784 |  |
| Headache due to intracranial disease | 512307 | 412307 | 784.0, 348.9 | R51.9, G93.9 |
| Recurrent occipital headache | 514036 | 414036 | 784 | R51.9 |
| Migraine variant without intractability | 515357 | 415357 | 346.2 | G43.809 |
| Migraine without aura and responsive to treatment | 515358 | 415358 | 346.1 | G43.009 |
| Tension headache NOS | 515502 | 415502 | 339.1 |  |
| Headache due to viral infection | 516578 | 416578 | 079.99, 784.0 | B34.9, R51.9 |
| Viral cephalgia | 516579 | 416579 | 079.99, 784.0 | B34.9, R51.9 |
| Headache due to viral infection | 516580 | 416580 | 784 |  |
| Viral cephalgia | 516581 | 416581 | 784 |  |
| Classical migraine without intractable migraine | 520241 | 420241 | 346 | G43.109 |
| Other forms of migraine | 521847 | 421847 | 346.8 | G43.809 |
| Headache | 522676 | 422676 | 784 | R51.9 |
| Unspecified migraine | 523334 | 423334 | 346.9 | G43.909 |
| Other headache syndromes | 523859 | 423859 | 339.89 | G44.89 |
| Drug induced headache, not elsewhere classified | 523860 | 423860 | 339.3, E980.5 | G44.40 |
| Other specified headache syndromes | 523861 | 423861 | 339.89 | G44.89 |
| Persistent migraine aura with cerebral infarction, without mention of intractable migraine without mention of status migrainosus | 523863 | 423863 | 346.60, 434.91 | G43.609, I63.9 |
| Persistent migraine aura with cerebral infarction, with intractable migraine, so stated, without mention of status migrainosus | 523864 | 423864 | 346.61, 434.91 | G43.619, I63.9 |
| Persistent migraine aura with cerebral infarction, without mention of intractable migraine with status migrainosus | 523865 | 423865 | 346.62, 434.91 | G43.601, I63.9 |
| Persistent migraine aura with cerebral infarction, with intractable migraine, so stated, with status migrainosus (HCC) | 523866 | 423866 | 346.63, 434.91 | G43.611, I63.9 |
| Cluster headaches and other trigeminal autonomic cephalgias | 523890 | 423890 | 339.00, 339.09 | G44.009, G44.099 |
| Variants of migraine, not elsewhere classified | 523891 | 423891 | 346.2 | G43.809 |
| Persistent migraine aura without cerebral infarction, intractable, with status migrainosus | 524521 | 424521 | 346.53 | G43.511 |
| Short lasting unilateral neuralgiform headache with conjunctival injection and tearing (SUNCT), intractable | 524523 | 424523 | 339.05 | G44.051 |
| Migraine without aura, not intractable | 524804 | 424804 | 346.1 | G43.009 |
| Chronic migraine without aura, not intractable | 524812 | 424812 | 346.7 | G43.709 |
| Migraine with aura, not intractable | 524822 | 424822 | 346 | G43.109 |
| Short lasting unilateral neuralgiform headache with conjunctival injection and tearing (SUNCT) | 524839 | 424839 | 339.05 | G44.059 |
| Intractable common transformed migraine without aura | 526302 | 426302 | 346.71 | G43.719 |
| Ocular migraine with status migrainosus, not intractable | 526404 | 426404 | 346.82 | G43.801 |
| Mixed common migraine and muscle contraction headache | 526582 | 426582 | 346.10, 307.81 | G43.009, G44.209 |
| Refractory basilar artery migraine | 526642 | 426642 | 346.01 | G43.119 |
| Non-refractory chronic migraine without aura | 526679 | 426679 | 346.7 | G43.709 |
| Ocular migraine with status migrainosus | 526894 | 426894 | 346.82 | G43.801 |
| Pregnancy headache in first trimester | 530104 | 430104 | 646.83, 784.0 | O26.891, R51.9 |
| Headache following intrapartum spinal anesthesia in second trimester | 530341 | 430341 | 668.81, 349.0 | O29.42 |
| Pregnancy headache in third trimester | 530518 | 430518 | 646.83, 784.0 | O26.893, R51.9 |
| Pregnancy headache in second trimester | 530538 | 430538 | 646.83, 784.0 | O26.892, R51.9 |
| Pregnancy headache in first trimester, antepartum | 530801 | 430801 | 646.83, 784.0 | O26.891, R51.9 |
| Pregnancy headache in third trimester, antepartum | 530877 | 430877 | 646.83, 784.0 | O26.893, R51.9 |
| Headache following intrapartum spinal anesthesia in first trimester | 531763 | 431763 | 668.81, 349.0 | O29.41 |
| Headache following intrapartum spinal anesthesia in third trimester | 531765 | 431765 | 668.81, 349.0 | O29.43 |
| Pregnancy headache in second trimester, antepartum | 531910 | 431910 | 646.83, 784.0 | O26.892, R51.9 |
| Frequent headaches | 580041 | 480041 | 784 | R51.9 |
| Nonintractable common migraine | 580122 | 480122 | 346.1 | G43.009 |
| Common migraine without intractability | 580123 | 480123 | 346.1 | G43.009 |
| Nonintractable migraine | 580125 | 480125 | 346.1 | G43.009 |
| Migraine without intractability | 580126 | 480126 | 346.1 | G43.009 |
| Migraine with aura and with status migrainosus | 583757 | 483757 | 346.02 | G43.101 |
| Persistent migraine aura with cerebral infarction and with status migrainosus (HCC) | 583771 | 483771 | 346.62, 434.91 | G43.601, I63.9 |
| Other forms of migraine with status migrainosus | 583804 | 483804 | 346.82 | G43.801 |
| Variants of migraine, not elsewhere classified, without intractable migraine with status migrainosus | 583874 | 483874 | 346.22 | G43.801 |
| Headache as manifestation of blood transfusion reaction | 583892 | 483892 | 999.89, 784.0, E879.8 | T80.89XA, R51.9 |
| Spinal and epidural anaesthesia-induced headache during pregnancy, delivered | 587241 | 487241 | 349 | O74.5 |
| Persistent migraine aura with cerebral infarction, with intractable migraine, so stated (HCC) | 587302 | 487302 | 346.61, 434.91 | G43.619, I63.9 |
| Variants of migraine, not elsewhere classified, with intractable migraine, so stated | 587315 | 487315 | 346.21 | G43.119 |
| Persistent migraine aura without cerebral infarction and with status migrainosus | 587488 | 487488 | 346.52 | G43.501 |
| Chronic right-sided headache | 590207 | 490207 | 784 | R51.9, G89.29 |
| Vestibular migraine | 590463 | 490463 | 346.8 | G43.809 |
| Discomfort of face | 590751 | 490751 | 784 | R51.9 |
| Facial discomfort | 591141 | 491141 | 784 | R51.9 |
| Intractable migraine equivalent | 591504 | 491504 | 346.81 | G43.119 |
| Intractable transformed migraine without aura and without status migrainosus | 593132 | 493132 | 346.71 | G43.719 |
| Migraine variant with headache | 594087 | 494087 | 346.2 | G43.809 |
| Migraine-cluster headache syndrome | 596549 | 496549 | 339 | G44.009 |
| Nonintractable headache | 596687 | 496687 | 784 | R51.9 |
| Episodic headache | 596746 | 496746 | 784 | R51.9 |
| Pressure and pain of right side of face | 599475 | 499475 | 784 | R51.9 |
| Spinal and epidural anesthesia-induced headache during pregnancy | 600362 | 500362 | 668.8 | O29.40 |
| Postpartum headache | 601116 | 501116 | 646.84, 784.0 | O90.89, R51.9 |
| Migrainous vertigo | 601772 | 501772 | 346.80, 780.4 | G43.109 |
| Postural headache | 604722 | 504722 | 784 | R51.0 |
| Severe migraine following anesthesia | 604782 | 504782 | 346.8 | T88.59XA, G43.809 |
| Familial basilar migraine | 606609 | 506609 | 346 | G43.109 |
| Familial hemiplegic migraine type 3 | 610266 | 510266 | 346.3 | G43.409 |
| Familial hemiplegic migraine type 2 | 610331 | 510331 | 346.3 | G43.409 |
| Familial hemiplegic migraine type 1 | 610335 | 510335 | 346.3 | G43.409 |
| Intractable chronic common migraine without aura | 612252 | 512252 | 346.71 | G43.719 |
| Intractable common migraine without aura | 614626 | 514626 | 346.11 | G43.019 |
| Spinal anesthesia-induced headache during labor and delivery | 619227 | 519227 | 668.8 | O74.5 |
| Epidural anesthesia-induced headache during labor and delivery | 619228 | 519228 | 668.8 | O74.5 |
| Spinal anesthesia-induced headache during puerperium | 619236 | 519236 | 668.84, 349.0 | O89.4 |
| Epidural anesthesia-induced headache during puerperium | 619237 | 519237 | 668.84, 349.0 | O89.4 |
| Headache syndrome | 619241 | 519241 | 339.89 | G44.89 |
| Intractable persistent migraine aura without cerebral infarction | 619242 | 519242 | 346.51 | G43.519 |
| Chronic intractable headache | 628081 | 528081 | 784 | R51.9, G89.29 |
| Acute intractable headache | 628082 | 528082 | 784 | R51.9 |
| Acute nonintractable headache | 628085 | 528085 | 784 | R51.9 |
| Chronic nonintractable headache | 628087 | 528087 | 784 | R51.9, G89.29 |
| Other headache syndrome | 732423 | 632423 | 339.89 | G44.89 |
| Spinal and epidural anesthesia induced headache during pregnancy, unspecified trimester | 732456 | 632456 | 668.83, 349.0 | O29.40 |
| Other migraine without status migrainosus, not intractable | 732520 | 632520 | 346.8 | G43.809 |
| Other migraine without status migrainosus, intractable | 732540 | 632540 | 346.81 | G43.819 |
| Other migraine with status migrainosus, not intractable | 732585 | 632585 | 346.82 | G43.801 |
| Other migraine with status migrainosus, intractable | 732586 | 632586 | 346.83 | G43.811 |
| Intractable drug-induced headache, not elsewhere classified | 732587 | 632587 | 339.3 | G44.41 |
| Drug-induced headache, not elsewhere classified, not intractable | 732588 | 632588 | 339.3, E980.5 | G44.40 |
| Other trigeminal autonomic cephalgia (TAC), not intractable | 732591 | 632591 | 339.09 | G44.099 |
| Other intractable trigeminal autonomic cephalgias (TAC) | 732592 | 632592 | 339.09 | G44.091 |
| Spinal and epidural anesthesia induced headache during pregnancy, third trimester | 732783 | 632783 | 646.83 | O29.43 |
| Spinal and epidural anesthesia induced headache during pregnancy, first trimester | 732804 | 632804 | 646.83 | O29.41 |
| Spinal and epidural anesthesia induced headache during pregnancy, second trimester | 732805 | 632805 | 646.83 | O29.42 |
| Other trigeminal autonomic cephalgias (tac), intractable | 734103 | 634103 | 339.09 | G44.091 |
| Headache in pregnancy, antepartum, second trimester | 742612 | 642612 | 646.83, 784.0 | O26.892, R51.9 |
| Headache in pregnancy, third trimester | 742914 | 642914 | 646.83, 784.0 | O26.893, R51.9 |
| Pregnancy headache, first trimester | 759008 | 659008 | 646.83, 784.0 | O26.891, R51.9 |
| Headache in pregnancy, first trimester | 761155 | 661155 | 646.83, 784.0 | O26.891, R51.9 |
| Pregnancy headache, antepartum, unspecified trimester | 762981 | 662981 | 646.83, 784.0 | O26.899, R51.9 |
| Post-procedural headache, subsequent encounter | 769854 | 669854 | V58.89, 784.0 | T81.89XD, R51.9 |
| Pregnancy headache, antepartum, first trimester | 777007 | 677007 | 646.83, 784.0 | O26.891, R51.9 |
| Pregnancy headache, unspecified trimester | 777078 | 677078 | 646.83, 784.0 | O26.899, R51.9 |
| Headache in pregnancy, antepartum, first trimester | 778924 | 678924 | 646.83, 784.0 | O26.891, R51.9 |
| Headache in pregnancy, antepartum, unspecified trimester | 779682 | 679682 | 646.83, 784.0 | O26.899, R51.9 |
| Post-procedural headache, initial encounter | 780059 | 680059 | 998.89, 784.0 | T81.89XA, R51.9 |
| Pregnancy headache, third trimester | 780795 | 680795 | 646.83, 784.0 | O26.893, R51.9 |
| Pregnancy headache, antepartum, second trimester | 784218 | 684218 | 646.83, 784.0 | O26.892, R51.9 |
| Headache in pregnancy, antepartum, third trimester | 784395 | 684395 | 646.83, 784.0 | O26.893, R51.9 |
| Pregnancy headache, second trimester | 786732 | 686732 | 646.83, 784.0 | O26.892, R51.9 |
| Headache in pregnancy, second trimester | 793292 | 693292 | 646.83, 784.0 | O26.892, R51.9 |
| Pregnancy headache, antepartum, third trimester | 795494 | 695494 | 646.83, 784.0 | O26.893, R51.9 |
| Headache in pregnancy, unspecified trimester | 796774 | 696774 | 646.83, 784.0 | O26.899, R51.9 |
| Other vascular headache | 806939 | 706939 | 784 | G44.1 |
| Headache following intrapartum spinal anesthesia, unspecified trimester | 993301 | 893301 | 668.80, 349.0 | O29.40 |
| Headache following intrapartum spinal anesthesia, second trimester | 993424 | 893424 | 668.81, 349.0 | O29.42 |
| Headache following intrapartum spinal anesthesia, first trimester | 993565 | 893565 | 668.81, 349.0 | O29.41 |
| Headache following intrapartum spinal anesthesia, third trimester | 993952 | 893952 | 668.81, 349.0 | O29.43 |
| Migraine without status migrainosus, not intractable, unspecified migraine type | 1139629 | 1039629 | 346.9 | G43.909 |
| Intractable migraine with status migrainosus, unspecified migraine type | 1139670 | 1039670 | 346.93 | G43.911 |
| Intractable tension-type headache, unspecified chronicity pattern | 1139701 | 1039701 | 339.1 | G44.201 |
| Cluster headache, not intractable, unspecified chronicity pattern | 1139716 | 1039716 | 339 | G44.009 |
| Migraine with status migrainosus, not intractable, unspecified migraine type | 1139737 | 1039737 | 346.92 | G43.901 |
| Tension-type headache, not intractable, unspecified chronicity pattern | 1139745 | 1039745 | 339.1 | G44.209 |
| Intractable cluster headache syndrome, unspecified chronicity pattern | 1139748 | 1039748 | 339 | G44.001 |
| Spinal and epidural anesthesia-induced headache during pregnancy, unspecified trimester | 1157901 | 1057901 | 668.83, 349.0 | O29.40 |
| Spinal and epidural anesthesia-induced headache during pregnancy, first trimester | 1167559 | 1067559 | 646.83 | O29.41 |
| Spinal and epidural anesthesia-induced headache during pregnancy, second trimester | 1176553 | 1076553 | 646.83 | O29.42 |
| Spinal and epidural anesthesia-induced headache during pregnancy, third trimester | 1177143 | 1077143 | 646.83 | O29.43 |
| Nonintractable migraine, unspecified migraine type | 1197603 | 1097603 | 346.1 | G43.009 |
| Headache, unspecified headache type | 1197845 | 1097845 | 784 | R51.9 |
| Chronic intractable headache, unspecified headache type | 1197862 | 1097862 | 784 | R51.9, G89.29 |
| Nonintractable headache, unspecified chronicity pattern, unspecified headache type | 1197876 | 1097876 | 784 | R51.9 |
| Intractable headache, unspecified chronicity pattern, unspecified headache type | 1197883 | 1097883 | 784 | R51.9 |
| Acute nonintractable headache, unspecified headache type | 1197998 | 1097998 | 784 | R51.9 |
| Chronic nonintractable headache, unspecified headache type | 1198009 | 1098009 | 784 | R51.9, G89.29 |
| Acute intractable headache, unspecified headache type | 1198019 | 1098019 | 784 | R51.9 |
| Other forms of migraine with intractable migraine, so stated | 1198109 | 1098109 | 346.81 | G43.819 |
| Migraine NEC/not intrcbl | 1198588 | 1098588 | 346.8 | G43.809 |
| Migraine NEC/intractable | 1198589 | 1098589 | 346.81 | G43.819 |
| Shrt lst uni nral hdache | 1200966 | 1100966 | 339.05 | G44.059 |
| Mgrn wo aura wo ntrc mgr | 1200967 | 1100967 | 346.12 | G43.001 |
| Var mgr NEC wo ntc mgr | 1200968 | 1100968 | 346.22 | G43.801 |
| Var mgrn NEC w ntrc mgr | 1200969 | 1100969 | 346.23 | G43.811 |
| Hmplg mgr wo ntrc wo st | 1200970 | 1100970 | 346.3 | G43.409 |
| Hmplg mgrn w ntrc wo st | 1200971 | 1100971 | 346.31 | G43.419 |
| Hemplg mgr wo ntrc w st | 1200972 | 1100972 | 346.32 | G43.401 |
| Hmplg mgrn w ntrc w st | 1200973 | 1100973 | 346.33 | G43.411 |
| Menst mgr wo ntrc wo st | 1200974 | 1100974 | 346.4 | G43.829 |
| Menstl mgrn w ntrc w st | 1200975 | 1100975 | 346.43 | G43.831 |
| Menstl mgr wo ntrc w st | 1200976 | 1100976 | 346.42 | G43.821 |
| Prst aura wo inf/ntr/st | 1200978 | 1100978 | 346.5 | G43.509 |
| Prs ara w ntr wo inf/st | 1200979 | 1100979 | 346.51 | G43.519 |
| Prs ara wo inf/ntr w st | 1200980 | 1100980 | 346.52 | G43.501 |
| Prs ara wo inf w ntr/st | 1200981 | 1100981 | 346.53 | G43.511 |
| Prs ara w inf wo ntr/st | 1200982 | 1100982 | 346.60, 434.91 | G43.609, I63.9 |
| Prs ara w/inf/ntr wo st | 1200983 | 1100983 | 346.61, 434.91 | G43.619, I63.9 |
| Prs ara wo ntr w inf/st | 1200984 | 1100984 | 346.62, 434.91 | G43.601, I63.9 |
| Prst ara w inf w ntr/st | 1200985 | 1100985 | 346.63, 434.91 | G43.611, I63.9 |
| Ch mgr wo ar wo nt wo st | 1200986 | 1100986 | 346.7 | G43.709 |
| Ch mgr wo ara w nt wo st | 1200987 | 1100987 | 346.71 | G43.719 |
| Ch mgr wo ara wo nt w st | 1200988 | 1100988 | 346.72 | G43.701 |
| Ch mgr wo ara w ntr w st | 1200989 | 1100989 | 346.73 | G43.711 |
| Mgr NOS wo ntrc w st mgr | 1200990 | 1100990 | 346.92 | G43.901 |
| Mgrn NOS w ntrc w st mgr | 1200991 | 1100991 | 346.93 | G43.911 |
| Menstl mgrn w ntrc wo st | 1201035 | 1101035 | 346.41 | G43.839 |
| Mgrn w aura wo ntrc mgrn | 1201038 | 1101038 | 346 | G43.109 |
| Mgrn w aura w intrc mgrn | 1201039 | 1101039 | 346.01 | G43.119 |
| Mgrn wo aura wo intrc mgr | 1201040 | 1101040 | 346.1 | G43.009 |
| Mgrn wo aura w intrc mgrn | 1201041 | 1101041 | 346.11 | G43.019 |
| Vrnt mgrn wo ntr mgr NEC | 1201042 | 1101042 | 346.2 | G43.109 |
| Vrnt mgrn w ntrc mgr NEC | 1201043 | 1101043 | 346.21 | G43.119 |
| Headache syndromes | 1201048 | 1101048 | 339.89 | G44.89 |
| Intractable variants of migraine with status migrainosus | 1201049 | 1101049 | 346.23 | G43.811 |
| Oth mgr w ntrc w st mgr | 1201511 | 1101511 | 346.83 | G43.811 |
| Oth mgr wo ntrc w st mgr | 1201512 | 1101512 | 346.82 | G43.801 |
| Variants of migraine, not elsewhere classified, with status migrainosus | 1202129 | 1102129 | 346.22 | G43.101 |
| Nonintractable chronic migraine | 1202444 | 1102444 | 346.7 | G43.909 |
| Migraine without status migrainosus | 1204925 | 1104925 | 346.9 | G43.909 |
| Persistent migraine aura with cerebral infarction without status migrainosus (HCC) | 1205065 | 1105065 | 346.6 | G43.609, I63.9 |
| Nonintractable paroxysmal hemicrania | 1205069 | 1105069 | 339.03 | G44.039 |
| Migraine without aura or status migrainosus | 1205072 | 1105072 | 346.1 | G43.009 |
| Nonintractable hemiplegic migraine | 1205075 | 1105075 | 346.3 | G43.409 |
| Persistent migraine aura without cerebral infarction or status migrainosus | 1205077 | 1105077 | 346.5 | G43.509 |
| Nonintractable persistent migraine aura without cerebral infarction | 1205088 | 1105088 | 346.5 | G43.509 |
| Nonintractable menstrual migraine | 1205105 | 1105105 | 346.4 | G43.829 |
| Menstrual migraine without status migrainosus | 1205107 | 1105107 | 346.4 | G43.829 |
| Hemiplegic migraine without status migrainosus | 1205108 | 1105108 | 346.3 | G43.409 |
| Chronic migraine without aura or status migrainosus | 1205109 | 1105109 | 346.7 | G43.709 |
| Intractable paroxysmal hemicrania | 1205119 | 1105119 | 339.03 | G44.031 |
| Intractable episodic headache | 1205122 | 1105122 | 784 | R51.9 |
| Nonintractable episodic headache | 1205129 | 1105129 | 784 | R51.9 |
| Intractable headache caused by drug | 1214560 | 1114560 | 339.3 | G44.41, T50.905A |
| Migraine due to estrogenic contraceptive | 1214698 | 1114698 | 346.90, E932.2 | G43.909, T38.4X5A |
| Intractable headache due to drug | 1214801 | 1114801 | 339.3 | G44.41, T50.905A |
| Acute tension-type headache | 1223488 | 1123488 | 339.1 | G44.209 |
| Acute tension headache | 1223503 | 1123503 | 339.1 | G44.209 |
| Acute intractable tension-type headache | 1223513 | 1123513 | 339.1 | G44.201 |
| Acute non intractable tension-type headache | 1223868 | 1123868 | 339.1 | G44.209 |
| Complicated migraine with status migrainosus | 1224080 | 1124080 | 346.02 | G43.101 |
| Intractable complicated migraine with status migrainosus | 1224091 | 1124091 | 346.93 | G43.111 |
| Migraine with aura and without status migrainosus | 1228761 | 1128761 | 346 | G43.109 |
| Lower-half facial migraine | 1234899 | 1134899 | 339 | G43.809 |
| Headache affecting lower half of face | 1234915 | 1134915 | 784 | R51.9 |
| Menstrual migraine, intractable, with status migrainosus | 1278855 | 1178855 | 346.43 | G43.831 |
| Migraine, unspecified, not intractable, without status migrainosus | 1279092 | 1179092 | 346.9 | G43.909 |
| Persistent migraine aura with cerebral infarction, not intractable, without status migrainosus (HCC) | 1279130 | 1179130 | 346.60, 434.91 | G43.609, I63.9 |
| Chronic paroxysmal hemicrania, intractable | 1279131 | 1179131 | 339.04 | G44.041 |
| Chronic migraine without aura, intractable, with status migrainosus | 1279476 | 1179476 | 346.73 | G43.711 |
| Hemiplegic migraine, intractable, with status migrainosus | 1279588 | 1179588 | 346.33 | G43.411 |
| Other migraine, intractable, without status migrainosus | 1279619 | 1179619 | 346.81 | G43.819 |
| Menstrual migraine, intractable, without status migrainosus | 1279710 | 1179710 | 346.41 | G43.839 |
| Menstrual migraine, not intractable, with status migrainosus | 1279715 | 1179715 | 346.42 | G43.821 |
| Migraine with aura, intractable, without status migrainosus | 1279716 | 1179716 | 346.01 | G43.119 |
| Persistent migraine aura without cerebral infarction, not intractable, without status migrainosus | 1279826 | 1179826 | 346.5 | G43.509 |
| Chronic cluster headache, intractable | 1280252 | 1180252 | 339.02 | G44.021 |
| Migraine without aura, not intractable, with status migrainosus | 1280470 | 1180470 | 346.12 | G43.001 |
| Drug-induced headache, not elsewhere classified, intractable | 1280513 | 1180513 | 339.3 | G44.41 |
| Migraine without aura, intractable, without status migrainosus | 1280847 | 1180847 | 346.11 | G43.019 |
| Episodic cluster headache, intractable | 1280882 | 1180882 | 339.01 | G44.011 |
| Other migraine, not intractable, without status migrainosus | 1281092 | 1181092 | 346.8 | G43.809 |
| Persistent migraine aura with cerebral infarction, intractable, without status migrainosus (HCC) | 1281127 | 1181127 | 346.61, 434.91 | G43.619, I63.9 |
| Chronic migraine without aura, not intractable, without status migrainosus | 1281478 | 1181478 | 346.7 | G43.709 |
| Persistent migraine aura without cerebral infarction, intractable, without status migrainosus | 1281599 | 1181599 | 346.51 | G43.519 |
| Migraine without aura, not intractable, without status migrainosus | 1281733 | 1181733 | 346.1 | G43.009 |
| Persistent migraine aura with cerebral infarction, not intractable, with status migrainosus (HCC) | 1281772 | 1181772 | 346.62, 434.91 | G43.601, I63.9 |
| Other migraine, intractable, with status migrainosus | 1282130 | 1182130 | 346.83 | G43.811 |
| Menstrual migraine, not intractable, without status migrainosus | 1282212 | 1182212 | 346.4 | G43.829 |
| Migraine, unspecified, not intractable, with status migrainosus | 1282590 | 1182590 | 346.92 | G43.901 |
| Other migraine, not intractable, with status migrainosus | 1282969 | 1182969 | 346.82 | G43.801 |
| Hemiplegic migraine, intractable, without status migrainosus | 1282973 | 1182973 | 346.31 | G43.419 |
| Tension-type headache, unspecified, not intractable | 1283617 | 1183617 | 339.1 | G44.209 |
| Tension-type headache, unspecified, intractable | 1283902 | 1183902 | 339.1 | G44.201 |
| Migraine with aura, not intractable, with status migrainosus | 1283989 | 1183989 | 346.03 | G43.101 |
| Persistent migraine aura without cerebral infarction, not intractable, with status migrainosus | 1284115 | 1184115 | 346.52 | G43.501 |
| Chronic migraine without aura, intractable, without status migrainosus | 1284246 | 1184246 | 346.71 | G43.719 |
| Cluster headache syndrome, unspecified, not intractable | 1284291 | 1184291 | 339 | G44.009 |
| Migraine with aura, not intractable, without status migrainosus | 1285379 | 1185379 | 346 | G43.109 |
| Episodic paroxysmal hemicrania, intractable | 1285427 | 1185427 | 339.03 | G44.031 |
| Cluster headache syndrome, unspecified, intractable | 1285944 | 1185944 | 339 | G44.001 |
| Persistent migraine aura with cerebral infarction, intractable, with status migrainosus (HCC) | 1286068 | 1186068 | 346.63, 434.91 | G43.611, I63.9 |
| Hemiplegic migraine, not intractable, without status migrainosus | 1286278 | 1186278 | 346.3 | G43.409 |
| Hemiplegic migraine, not intractable, with status migrainosus | 1286411 | 1186411 | 346.32 | G43.401 |
| Other trigeminal autonomic cephalgias (tac), not intractable | 1286430 | 1186430 | 339.09 | G44.099 |
| Chronic migraine without aura, not intractable, with status migrainosus | 1286511 | 1186511 | 346.72 | G43.701 |
| Migraine, unspecified, intractable, with status migrainosus | 1286513 | 1186513 | 346.93 | G43.911 |
| Other reaction to spinal and lumbar puncture | 1292355 | 1192355 | 349 | G97.1 |
| Spinal and epidural anesthesia-induced headache during the puerperium | 1292652 | 1192652 | 668.84, 349.0 | O89.4 |
| Periodic headache syndromes in child or adult, not intractable | 1294577 | 1194577 | 346.2 | G43.C0 |
| Periodic headache syndromes in child or adult, intractable | 1294579 | 1194579 | 346.21 | G43.C1 |
| Vascular headache, not elsewhere classified | 1295085 | 1195085 | 784 | G44.1 |
| Other drug induced headache | 1299976 | 1199976 | 339.3, E980.5 | G44.40 |
| Other intractable trigeminal autonomic cephalgia (TAC) | 1299977 | 1199977 | 339.09 | G44.091 |
| Other type of intractable migraine | 1299978 | 1199978 | 346.81 | G43.819 |
| Other type of nonintractable migraine | 1299979 | 1199979 | 346.8 | G43.809 |
| Other type of migraine | 1299980 | 1199980 | 346.8 | G43.809 |
| Other type of migraine without status migrainosus | 1299986 | 1199986 | 346.8 | G43.809 |
| Other trigeminal autonomic cephalgia (TAC) | 1300020 | 1200020 | 339.09 | G44.099 |
| Nonintractable episodic headache, unspecified headache type | 1335761 | 1235761 | 784 | R51.9 |
| Nonintractable paroxysmal hemicrania, unspecified chronicity pattern | 1336124 | 1236124 | 339.03 | G44.039 |
| Intractable episodic headache, unspecified headache type | 1336127 | 1236127 | 784 | R51.9 |
| Intractable paroxysmal hemicrania, unspecified chronicity pattern | 1336250 | 1236250 | 339.03 | G44.031 |
| Intermittent headache | 1359648 | 1259648 | 784 | R51.9 |
| Periodic migrainous neuralgia | 1360052 | 1260052 | 339 | G44.009 |
| Chronic left-sided headache | 1360920 | 1260920 | 784 | R51.9, G89.29 |
| Facial pain of nontraumatic and non-dental origin | 1365149 | 1265149 | 784 |  |
| Persistent migraine aura with cerebral infarction, not intractable, with status migrainosus (CODE) | 1383030 | 1283030 | 346.62, 434.91 | G43.601 |
| Persistent migraine aura with cerebral infarction, not intractable, without status migrainosus (CODE) | 1383031 | 1283031 | 346.60, 434.91 | G43.609 |
| Persistent migraine aura with cerebral infarction, intractable, with status migrainosus (CODE) | 1383032 | 1283032 | 346.63, 434.91 | G43.611 |
| Persistent migraine aura with cerebral infarction, intractable, without status migrainosus (CODE) | 1383033 | 1283033 | 346.61, 434.91 | G43.619 |
| Headache associated with menopause | 1400839 | 1300839 | 784.0, 627.2 | N95.1, R51.9 |
| Retinal status migrainosus | 1406121 | 1306121 | 346.02 | G43.101 |
| Headache due to oral contraceptive | 1406920 | 1306920 | 339.3, E932.2 |  |
| Parietal headache | 1407172 | 1307172 | 784 |  |
| Other headache syndromes | 1448195 | 1348195 | 339.89 | G44.89 |
| Drug induced headache, not elsewhere classified | 1448196 | 1348196 | 339.3, E980.5 | G44.40 |
| Persistent migraine aura with cerebral infarction, without mention of intractable migraine without mention of status migrainosus | 1448197 | 1348197 | 346.60, 434.91 | G43.609, I63.9 |
| Persistent migraine aura with cerebral infarction, with intractable migraine, so stated, without mention of status migrainosus | 1448198 | 1348198 | 346.61, 434.91 | G43.619, I63.9 |
| Persistent migraine aura with cerebral infarction, without mention of intractable migraine with status migrainosus | 1448199 | 1348199 | 346.62, 434.91 | G43.601, I63.9 |
| Headache after spinal puncture | 1452247 | 1352247 | 349 | G97.1 |
| Headache due to anesthesia | 1452248 | 1352248 | 784 | T88.59XA, R51.9 |
| Shrt lst unil nerlgif hdache w cnjnct inject/tear, ntrct | 1465537 | 1365537 | 339.05 | G44.051 |
| Perst migraine aura w cereb infrc, not ntrct, w/o stat migr (HCC) | 1465551 | 1365551 | 346.60, 434.91 | G43.609, I63.9 |
| Spinal and epidur anesth induce hdache dur preg, third tri | 1465668 | 1365668 | 646.83 | O29.43 |
| Spinal and epidur anesth induce hdache during preg, unsp tri | 1465848 | 1365848 | 668.83, 349.0 | O29.40 |
| Perst migraine aura w cerebral infrc, not ntrct, w stat migr (HCC) | 1465883 | 1365883 | 346.62, 434.91 | G43.601, I63.9 |
| Spinal and epidur anesth induce hdache dur preg, second tri | 1465945 | 1365945 | 646.83 | O29.42 |
| Perst migraine aura w cerebral infrc, ntrct, w/o stat migr (HCC) | 1466063 | 1366063 | 346.61, 434.91 | G43.619, I63.9 |
| Spinal and epidur anesth induce hdache dur preg, first tri | 1466120 | 1366120 | 646.83 | O29.41 |
| Perst migraine aura w/o cereb infrc, not ntrct, w stat migr | 1466156 | 1366156 | 346.52 | G43.501 |
| Perst migraine aura w cerebral infrc, ntrct, w stat migr (HCC) | 1466498 | 1366498 | 346.63, 434.91 | G43.611, I63.9 |
| Chronic migraine w/o aura, not intractable, w stat migr | 1466673 | 1366673 | 346.72 | G43.701 |
| Chronic migraine w/o aura, not intractable, w/o stat migr | 1466729 | 1366729 | 346.7 | G43.709 |
| Perst migraine aura w/o cerebral infrc, ntrct, w stat migr | 1466829 | 1366829 | 346.53 | G43.511 |
| Perst migraine aura w/o cerebral infrc, ntrct, w/o stat migr | 1466830 | 1366830 | 346.51 | G43.519 |
| Perst migrn aura w/o cereb infrc, not ntrct, w/o stat migr | 1466970 | 1366970 | 346.5 | G43.509 |
| Shrt lst unil nerlgif hdache w cnjnct inject/tear, not ntrct | 1467131 | 1367131 | 339.05 | G44.059 |
| Spinal and epidural anaesthesia-induced headache during pregnancy, antepartum | 1468422 | 1368422 | 668.83, 349.0 | O29.40 |
| Vascular headache, not elsewhere classified (CODE) | 1528295 | 1428295 | 784 | G44.1 |
| Stroke with migraine (HCC) | 1530103 | 1430103 | 434.91, 346.90 | I63.9, G43.909 |
| Drug-induced headache | 1531026 | 1431026 | 339.3, E980.5 | G44.40 |
| Classical migraine with status migrainosus | 1531272 | 1431272 | 346.02 | G43.101 |
| Headache due to low cerebrospinal fluid pressure | 1532525 | 1432525 | 784 | R51.0 |
| Nitroglycerin-induced headache | 1539267 | 1439267 | 339.3, E942.4 | G44.40, T46.3X5A |
| Chronic primary headache | 1540060 | 1440060 | 784 | R51.9, G89.29 |
| Chronic secondary facial pain | 1540503 | 1440503 | 784.0, 338.29 | R51.9, G89.29 |
| Headache in pediatric patient | 1543140 | 1443140 | 784 | R51.9 |
| Transdermal nitroglycerin-induced headache | 1543256 | 1443256 | 339.3, E942.4 | G44.40, T46.3X5A |
| Classical migraine with intractable migraine with aura | 1543261 | 1443261 | 346.01 | G43.119 |
| Dialysis headache | 1547482 | 1447482 | 339.89, E879.1 | G44.89, Y84.1 |
| Chronic migraine with aura | 1547751 | 1447751 | 346 | G43.109 |
| Complaint of headache | 1549119 | 1449119 | 784 | R51.9 |
| Sudden onset unilateral headache | 1552429 | 1452429 | 784 | R51.9 |
| Acute headache with normal neurologic examination | 1552463 | 1452463 | 784 | R51.9 |
| New onset of headache in pregnant patient | 1552505 | 1452505 | 646.80, 784.0 | O26.899, R51.9 |
| Chronic headache with normal neurologic examination | 1552565 | 1452565 | 784 | R51.9, G89.29 |
| Nonintractable cyclical vomiting with status migrainosus | 1553536 | 1453536 | 346.22 | G43.A0, G43.901 |
| Cyclical vomiting associated with nonintractable migraine | 1553537 | 1453537 | 346.2 | G43.A0 |
| Cyclical vomiting associated with migraine, intractability of vomiting not specified | 1555094 | 1455094 | 346.2 | G43.A0 |
| Cyclical vomiting, in migraine, not intractable | 1556011 | 1456011 | 346.2 | G43.A0 |
| Spin/ep anaes ind headache preg/deliv | 1557708 | 1457708 | 349 |  |
| Spin/ep anaes ind headache preg/antepart | 1558003 | 1458003 | 668.83, 349.0 |  |
| Nummular headache | 1558805 | 1458805 | 339.89 | G44.89 |
| Chronic idiopathic facial pain | 1559340 | 1459340 | 784.0, 338.29 | R51.9, G89.29 |
| Intracranial hypotension due to and not concurrent with lumbar puncture | 1561689 | 1461689 | 349.0, 458.8 | G97.84 |
| Headache with remote history of traumatic head injury | 1562868 | 1462868 | 784.0, V15.59 | R51.9, Z87.828 |
| Chronic headache with new features | 1562894 | 1462894 | 784 | R51.9, G89.29 |
| Headache associated with infection | 1562990 | 1462990 | 136.9, 784.0 | B99.9, R51.9 |
| Chronic headache without new features | 1563130 | 1463130 | 784 | R51.9, G89.29 |
| Headache with neurologic deficit | 1563192 | 1463192 | 784.0, 781.99 | R51.9, R29.818 |
| Headache, unspecified | 1564740 | 1464740 | 784 | R51.9 |
| Headache with orthostatic component, not elsewhere classified | 1565967 | 1465967 | 784 | R51.0 |
| Headache after vaccination | 1569334 | 1469334 | 339.3, E949.9 | G44.40, T50.Z95A |
| Transformed migraine | 1572088 | 1472088 | 346.7 | G43.709 |
| Migraine with persistent visual aura | 1572321 | 1472321 | 346.5 | G43.509 |
| Headache due to high altitude | 1572819 | 1472819 | 784.0, 993.2 | R51.9, T70.29XA |
| Post-COVID-19 syndrome manifesting as chronic headache | 1573487 | 1473487 | 784.0, 139.8, 338.29 | R51.9, U09.9, G89.29 |
| COVID-19 long hauler manifesting chronic headache | 1573488 | 1473488 | 784.0, 139.8, 338.29 | R51.9, U09.9, G89.29 |
| Post-COVID chronic headache | 1573564 | 1473564 | 784.0, 139.8, 338.29 | R51.9, U09.9, G89.29 |
| Headache after cough | 1574147 | 1474147 | 339.83 | G44.83 |
| Migraine aura occurring with and without headache | 1576694 | 1476694 | 346 | G43.109 |
| Acephalgic migraine | 1576721 | 1476721 | 346 | G43.109 |
| Acute migraine | 1578100 | 1478100 | 346.9 | G43.909 |
| Episodic migraine | 1578128 | 1478128 | 346.9 | G43.909 |
| Epicrania fugax | 1578753 | 1478753 | 339.89 | G44.89 |
| Headache due to temporomandibular disorder | 1580339 | 1480339 | 784.0, 524.60 | R51.9, M26.609 |
| Cyclical vomiting associated with migraine, unspecified whether migraine intractable | 1582638 | 1482638 | 346.2 | G43.A0 |
| Tension headache | 307.81.ICD-9-CM | 186121 | 307.81 | G44.209 |
| Cluster headache syndrome, unspecified | 339.00.ICD-9-CM | 185324 | 339 | G44.009 |
| Episodic cluster headache | 339.01.ICD-9-CM | 185325 | 339.01 | G44.019 |
| Chronic cluster headache | 339.02.ICD-9-CM | 185327 | 339.02 | G44.029 |
| Episodic paroxysmal hemicrania | 339.03.ICD-9-CM | 17630 | 339.03 | G44.039 |
| Chronic paroxysmal hemicrania | 339.04.ICD-9-CM | 185328 | 339.04 | G44.049 |
| Short lasting unilateral neuralgiform headache with conjunctival injection and tearing | 339.05.ICD-9-CM | 185596 | 339.05 | G44.059 |
| Other trigeminal autonomic cephalgias | 339.09.ICD-9-CM | 17633 | 339.09 | G44.099 |
| Tension type headache, unspecified | 339.10.ICD-9-CM | 185330 | 339.1 | G44.209 |
| Episodic tension type headache | 339.11.ICD-9-CM | 185331 | 339.11 | G44.219 |
| Chronic tension type headache | 339.12.ICD-9-CM | 185332 | 339.12 | G44.229 |
| Drug induced headache, not elsewhere classified(339.3) | 339.3.ICD-9-CM | 17640 | 339.3 | G44.40 |
| Hypnic headache | 339.81.ICD-9-CM | 185608 | 339.81 | G44.81 |
| Headache associated with sexual activity | 339.82.ICD-9-CM | 17646 | 339.82 | G44.82 |
| Primary cough headache | 339.83.ICD-9-CM | 185348 | 339.83 | G44.83 |
| Primary exertional headache | 339.84.ICD-9-CM | 185350 | 339.84 | G44.84 |
| Primary stabbing headache | 339.85.ICD-9-CM | 185352 | 339.85 | G44.85 |
| Other headache syndromes(339.89) | 339.89.ICD-9-CM | 17650 | 339.89 | G44.89 |
| Migraine with aura, without mention of intractable migraine without mention of status migrainosus | 346.00.ICD-9-CM | 186413 | 346 | G43.109 |
| Migraine with aura, with intractable migraine, so stated, without mention of status migrainosus | 346.01.ICD-9-CM | 186423 | 346.01 | G43.119 |
| Migraine with aura, without mention of intractable migraine with status migrainosus | 346.02.ICD-9-CM | 185613 | 346.02 | G43.101 |
| Migraine with aura, with intractable migraine, so stated, with status migrainosus | 346.03.ICD-9-CM | 185614 | 346.03 | G43.111 |
| Migraine without aura, without mention of intractable migraine without mention of status migrainosus | 346.10.ICD-9-CM | 186424 | 346.1 | G43.009 |
| Migraine without aura, with intractable migraine, so stated, without mention of status migrainosus | 346.11.ICD-9-CM | 186425 | 346.11 | G43.019 |
| Migraine without aura, without mention of intractable migraine with status migrainosus | 346.12.ICD-9-CM | 185615 | 346.12 | G43.001 |
| Migraine without aura, with intractable migraine, so stated, with status migrainosus | 346.13.ICD-9-CM | 185616 | 346.13 | G43.011 |
| Variants of migraine, not elsewhere classified, without mention of intractable migraine without mention of status migrainosus | 346.20.ICD-9-CM | 186435 | 346.2 | G43.809 |
| Variants of migraine, not elsewhere classified, with intractable migraine, so stated, without mention of status migrainosus | 346.21.ICD-9-CM | 186436 | 346.21 | G43.119 |
| Variants of migraine, not elsewhere classified, without mention of intractable migraine with status migrainosus | 346.22.ICD-9-CM | 185617 | 346.22 | G43.101 |
| Variants of migraine, not elsewhere classified, with intractable migraine, so stated, with status migrainosus | 346.23.ICD-9-CM | 185618 | 346.23 | G43.811 |
| Hemiplegic migraine, without mention of intractable migraine without mention of status migrainosus | 346.30.ICD-9-CM | 185619 | 346.3 | G43.409 |
| Hemiplegic migraine, with intractable migraine, so stated, without mention of status migrainosus | 346.31.ICD-9-CM | 185620 | 346.31 | G43.419 |
| Hemiplegic migraine, without mention of intractable migraine with status migrainosus | 346.32.ICD-9-CM | 185621 | 346.32 | G43.401 |
| Hemiplegic migraine, with intractable migraine, so stated, with status migrainosus | 346.33.ICD-9-CM | 185622 | 346.33 | G43.411 |
| Menstrual migraine, without mention of intractable migraine without mention of status migrainosus | 346.40.ICD-9-CM | 185623 | 346.4 | G43.829 |
| Menstrual migraine, with intractable migraine, so stated, without mention of status migrainosus | 346.41.ICD-9-CM | 186101 | 346.41 | G43.839 |
| Menstrual migraine, without mention of intractable migraine with status migrainosus | 346.42.ICD-9-CM | 185625 | 346.42 | G43.821 |
| Menstrual migraine, with intractable migraine, so stated, with status migrainosus | 346.43.ICD-9-CM | 185624 | 346.43 | G43.831 |
| Persistent migraine aura without cerebral infarction, without mention of intractable migraine without mention of status migrainosus | 346.50.ICD-9-CM | 185719 | 346.5 | G43.509 |
| Persistent migraine aura without cerebral infarction, with intractable migraine, so stated, without mention of status migrainosus | 346.51.ICD-9-CM | 185720 | 346.51 | G43.519 |
| Persistent migraine aura without cerebral infarction, without mention of intractable migraine with status migrainosus | 346.52.ICD-9-CM | 185721 | 346.52 | G43.501 |
| Persistent migraine aura without cerebral infarction, with intractable migraine, so stated, with status migrainosus | 346.53.ICD-9-CM | 185722 | 346.53 | G43.511 |
| Persistent migraine aura with cerebral infarction, without mention of intractable migraine without mention of status migrainosus(346.60) | 346.60.ICD-9-CM | 185723 | 346.6 | G43.609, I63.9 |
| Persistent migraine aura with cerebral infarction, with intractable migraine, so stated, without mention of status migrainosus(346.61) | 346.61.ICD-9-CM | 185724 | 346.61 | G43.619, I63.9 |
| Persistent migraine aura with cerebral infarction, without mention of intractable migraine with status migrainosus(346.62) | 346.62.ICD-9-CM | 185726 | 346.62 | G43.601, I63.9 |
| Persistent migraine aura with cerebral infarction, with intractable migraine, so stated, with status migrainosus(346.63) | 346.63.ICD-9-CM | 185727 | 346.63 | G43.611, I63.9 |
| Chronic migraine without aura, without mention of intractable migraine without mention of status migrainosus | 346.70.ICD-9-CM | 185728 | 346.7 | G43.709 |
| Chronic migraine without aura, with intractable migraine, so stated, without mention of status migrainosus | 346.71.ICD-9-CM | 185729 | 346.71 | G43.719 |
| Chronic migraine without aura, without mention of intractable migraine with status migrainosus | 346.72.ICD-9-CM | 185730 | 346.72 | G43.701 |
| Chronic migraine without aura, with intractable migraine, so stated, with status migrainosus | 346.73.ICD-9-CM | 185731 | 346.73 | G43.711 |
| Other forms of migraine, without mention of intractable migraine without mention of status migrainosus | 346.80.ICD-9-CM | 3592 | 346.8 | G43.809 |
| Other forms of migraine, with intractable migraine, so stated, without mention of status migrainosus | 346.81.ICD-9-CM | 3593 | 346.81 | G43.819 |
| Other forms of migraine, without mention of intractable migraine with status migrainosus | 346.82.ICD-9-CM | 17677 | 346.82 | G43.801 |
| Other forms of migraine, with intractable migraine, so stated, with status migrainosus | 346.83.ICD-9-CM | 17678 | 346.83 | G43.811 |
| Migraine, unspecified, without mention of intractable migraine without mention of status migrainosus | 346.90.ICD-9-CM | 83958 | 346.9 | G43.909 |
| Migraine, unspecified, without mention of intractable migraine with status migrainosus | 346.92.ICD-9-CM | 185734 | 346.92 | G43.901 |
| Migraine, unspecified, with intractable migraine, so stated, with status migrainosus | 346.93.ICD-9-CM | 185735 | 346.93 | G43.911 |
| Reaction to spinal or lumbar puncture | 349.0.ICD-9-CM | 61432 | 349 | G97.1 |
| Headache(784.0) | 784.0.ICD-9-CM | 9781 | 784 | R51 |
| Migraine without aura, not intractable, with status migrainosus | G43.001 | 596574 |  | G43.001 |
| Migraine without aura, not intractable, without status migrainosus | G43.009 | 598055 |  | G43.009 |
| Migraine without aura, intractable, with status migrainosus | G43.011 | 587414 |  | G43.011 |
| Migraine without aura, intractable, without status migrainosus | G43.019 | 597016 |  | G43.019 |
| Migraine with aura, not intractable, with status migrainosus | G43.101 | 600680 |  | G43.101 |
| Migraine with aura, not intractable, without status migrainosus | G43.109 | 602319 |  | G43.109 |
| Migraine with aura, intractable, with status migrainosus | G43.111 | 587412 |  | G43.111 |
| Migraine with aura, intractable, without status migrainosus | G43.119 | 595697 |  | G43.119 |
| Hemiplegic migraine, not intractable, with status migrainosus | G43.401 | 603504 |  | G43.401 |
| Hemiplegic migraine, not intractable, without status migrainosus | G43.409 | 603349 |  | G43.409 |
| Hemiplegic migraine, intractable, with status migrainosus | G43.411 | 595554 |  | G43.411 |
| Hemiplegic migraine, intractable, without status migrainosus | G43.419 | 599501 |  | G43.419 |
| Persistent migraine aura without cerebral infarction, not intractable, with status migrainosus | G43.501 | 600825 |  | G43.501 |
| Persistent migraine aura without cerebral infarction, not intractable, without status migrainosus | G43.509 | 595829 |  | G43.509 |
| Persistent migraine aura without cerebral infarction, intractable, with status migrainosus | G43.511 | 597477 |  | G43.511 |
| Persistent migraine aura without cerebral infarction, intractable, without status migrainosus | G43.519 | 597910 |  | G43.519 |
| Persistent migraine aura with cerebral infarction, not intractable, with status migrainosus | G43.601 | 598097 |  | G43.601 |
| Persistent migraine aura with cerebral infarction, not intractable, without status migrainosus | G43.609 | 595025 |  | G43.609 |
| Persistent migraine aura with cerebral infarction, intractable, with status migrainosus | G43.611 | 603109 |  | G43.611 |
| Persistent migraine aura with cerebral infarction, intractable, without status migrainosus | G43.619 | 597358 |  | G43.619 |
| Chronic migraine without aura, not intractable, with status migrainosus | G43.701 | 603626 |  | G43.701 |
| Chronic migraine without aura, not intractable, without status migrainosus | G43.709 | 597771 |  | G43.709 |
| Chronic migraine without aura, intractable, with status migrainosus | G43.711 | 595426 |  | G43.711 |
| Chronic migraine without aura, intractable, without status migrainosus | G43.719 | 600979 |  | G43.719 |
| Other migraine, not intractable, with status migrainosus | G43.801 | 599497 |  | G43.801 |
| Other migraine, not intractable, without status migrainosus | G43.809 | 597316 |  | G43.809 |
| Other migraine, intractable, with status migrainosus | G43.811 | 598516 |  | G43.811 |
| Other migraine, intractable, without status migrainosus | G43.819 | 595591 |  | G43.819 |
| Menstrual migraine, not intractable, with status migrainosus | G43.821 | 595695 |  | G43.821 |
| Menstrual migraine, not intractable, without status migrainosus | G43.829 | 598614 |  | G43.829 |
| Menstrual migraine, intractable, with status migrainosus | G43.831 | 594701 |  | G43.831 |
| Menstrual migraine, intractable, without status migrainosus | G43.839 | 595689 |  | G43.839 |
| Migraine, unspecified, not intractable, with status migrainosus | G43.901 | 599068 |  | G43.901 |
| Migraine, unspecified, not intractable, without status migrainosus | G43.909 | 594985 |  | G43.909 |
| Migraine, unspecified, intractable, with status migrainosus | G43.911 | 603628 |  | G43.911 |
| Periodic headache syndromes in child or adult, not intractable | G43.C0 | 610940 |  | G43.C0 |
| Periodic headache syndromes in child or adult, intractable | G43.C1 | 610942 |  | G43.C1 |
| Cluster headache syndrome, unspecified, intractable | G44.001 | 602968 |  | G44.001 |
| Cluster headache syndrome, unspecified, not intractable | G44.009 | 601030 |  | G44.009 |
| Episodic cluster headache, intractable | G44.011 | 597056 |  | G44.011 |
| Episodic cluster headache, not intractable | G44.019 | 589608 |  | G44.019 |
| Chronic cluster headache, intractable | G44.021 | 596323 |  | G44.021 |
| Chronic cluster headache, not intractable | G44.029 | 589607 |  | G44.029 |
| Episodic paroxysmal hemicrania, intractable | G44.031 | 602373 |  | G44.031 |
| Episodic paroxysmal hemicrania, not intractable | G44.039 | 589563 |  | G44.039 |
| Chronic paroxysmal hemicrania, intractable | G44.041 | 595026 |  | G44.041 |
| Chronic paroxysmal hemicrania, not intractable | G44.049 | 589562 |  | G44.049 |
| Short lasting unilateral neuralgiform headache with conjunctival injection and tearing (SUNCT), intractable | G44.051 | 598659 |  | G44.051 |
| Short lasting unilateral neuralgiform headache with conjunctival injection and tearing (SUNCT), not intractable | G44.059 | 589561 |  | G44.059 |
| Other trigeminal autonomic cephalgias (tac), intractable | G44.091 | 602065 |  | G44.091 |
| Other trigeminal autonomic cephalgias (tac), not intractable | G44.099 | 603527 |  | G44.099 |
| Vascular headache, not elsewhere classified | G44.1 | 611551 |  | G44.1 |
| Tension-type headache, unspecified, intractable | G44.201 | 600571 |  | G44.201 |
| Tension-type headache, unspecified, not intractable | G44.209 | 600241 |  | G44.209 |
| Episodic tension-type headache, intractable | G44.211 | 540538 |  | G44.211 |
| Episodic tension-type headache, not intractable | G44.219 | 589619 |  | G44.219 |
| Chronic tension-type headache, intractable | G44.221 | 538952 |  | G44.221 |
| Chronic tension-type headache, not intractable | G44.229 | 538951 |  | G44.229 |
| Hypnic headache | G44.81 | 538849 |  | G44.81 |
| Headache associated with sexual activity | G44.82 | 538850 |  | G44.82 |
| Primary cough headache | G44.83 | 538851 |  | G44.83 |
| Primary exertional headache | G44.84 | 538852 |  | G44.84 |
| Primary stabbing headache | G44.85 | 538853 |  | G44.85 |
| Other headache syndrome | G44.89 | 587224 |  | G44.89 |
| Spinal and epidural anesthesia induced headache during pregnancy, unspecified trimester | O29.40 | 588028 |  | O29.40 |
| Spinal and epidural anesthesia induced headache during pregnancy, first trimester | O29.41 | 591272 |  | O29.41 |
| Spinal and epidural anesthesia induced headache during pregnancy, second trimester | O29.42 | 591273 |  | O29.42 |
| Spinal and epidural anesthesia induced headache during pregnancy, third trimester | O29.43 | 591106 |  | O29.43 |
| Spinal and epidural anesthesia-induced headache during labor and delivery | O74.5 | 540657 |  | O74.5 |
| Spinal and epidural anesthesia-induced headache during the puerperium | O89.4 | 609515 |  | O89.4 |
| Headache | R51 | 535977 |  | R51 |

***Exclusion Criteria***

- G44.3 Post-traumatic headache
- G45.0 Vertebro-basilar artery syndrome
- G45.1 Carotid artery syndrome (hemispheric)
- G45.2 Multiple and bilateral precerebral artery syndromes
- G45.3 Amaurosis fugax
- G45.4 Transient global amnesia
- G45.8 Other transient cerebral ischemic attacks and related syndromes
- G45.9 Transient cerebral ischemic attack, unspecified
- G46.0 Middle cerebral artery syndrome
- G46.1 Anterior cerebral artery syndrome
- G46.2 Posterior cerebral artery syndrome
- G46.3 Brain stem stroke syndrome
- G46.4 Cerebellar stroke syndrome
- G46.5 Pure motor lacunar syndrome
- G46.6 Pure sensory lacunar syndrome
- G46.7 Other lacunar syndromes
- G46.8 Other vascular syndromes of brain in cerebrovascular diseases
- J01.90 Acute Sinusitis, unspecified
- I63.6 Cerebral infarction due to cerebral venous thrombosis, nonpyogenic
- J32.9 Chronic sinusitis
- I67.1 Cerebral aneurysm, nonruptured
- C71.9 Malignant neoplasm of brain, unspecified
- Q070 Arnold-Chiari malformation
- S06.0- Concussion
- S06.1- Traumatic cerebral edema
- S06.2- Diffuse traumatic brain injury
- S06.3- Focal traumatic brain injury
- S06.4- Epidural hemorrhage
- S06.5- Traumatic subdural hemorrhage
- S06.6- Traumatic subarachnoid hemorrhage
- S06.8- Other specified intracranial injuries
- S06.9- Unspecified intracranial injury
- S06.A- Traumatic brain compression and herniation
- U07.1 Coronavirus
- H65.0 Acute serous otitis media
- H65.1 Other acute nonsuppurative otitis media
- H65.2 Chronic serous otitis media
- H65.3 Chronic mucoid otitis media
- H65.4 Other chronic nonsuppurative otitis media
- H65.9 Unspecified nonsuppurative otitis media
- H66.0 Acute suppurative otitis media
- H66.1 Chronic tubotympanic suppurative otitis media
- H66.2 Chronic atticoantral suppurative otitis media
- H66.3 Other chronic suppurative otitis media
- H66.4 Suppurative otitis media, unspecified
- H66.9 Otitis media, unspecified
- H67.1 Otitis media in diseases classified elsewhere, right ear
- H67.2
- Otitis media in diseases classified elsewhere, left ear
- H67.3 Otitis media in diseases classified elsewhere, bilateral
- H67.9 Otitis media in diseases classified elsewhere, unspecified ear
- G04.0, G04.00, 01,02 Acute disseminated encephalitis and encephalomyelitis (ADEM
- G04.2 Bacterial meningoencephalitis and meningomyelitis, not elsewhere classified
- G04.3 (G04.30, 31, 32, 39) Acute necrotizing hemorrhagic encephalopathy
- G04.8 (G04.81, 82, 89) Other encephalitis, myelitis and encephalomyelitis
- G04.9 (G04.90, 91) Encephalitis, myelitis and encephalomyelitis, unspecified
- I67.4 Hypertensive encephalopathy
- O14 (O14.0, 1, 2, 9) Pre-eclampsia
- O15.0 Eclampsia complicating pregnancy
- O15.00 Eclampsia complicating pregnancy, unspecified trimester
- O15.02 Eclampsia complicating pregnancy, second trimester
- O15.03 Eclampsia complicating pregnancy, third trimester
- O15.1 Eclampsia complicating labor
- O15.2 Eclampsia complicating the puerperium
- O15.9 Eclampsia, unspecified as to time period
- I16.0 Hypertensive urgency
- I16.1 Hypertensive emergency
- I16.9 Hypertensive crisis, unspecified
- J09.X Influenza due to identified novel influenza A virus
  - J09.X1 …… with pneumonia
  - J09.X2 …… with other respiratory manifestations
  - J09.X3 …… with gastrointestinal manifestations
  - J09.X9 …… with other manifestations
- J10.0 Influenza due to other identified influenza virus with pneumonia
  - J10.00 Influenza due to other identified influenza virus with unspecified type of pneumonia
  - J10.01 Influenza due to other identified influenza virus with the same other identified influenza virus pneumonia
  - J10.08 Influenza due to other identified influenza virus with other specified pneumonia
- J10.1 Influenza due to other identified influenza virus with other respiratory manifestations
- J10.2 Influenza due to other identified influenza virus with gastrointestinal manifestations
- J10.8 Influenza due to other identified influenza virus with other manifestations
  - J10.81 Influenza due to other identified influenza virus with encephalopathy
  - J10.82 Influenza due to other identified influenza virus with myocarditis
  - J10.83 Influenza due to other identified influenza virus with otitis media
  - J10.89 Influenza due to other identified influenza virus with other manifestations
- J11.0 Influenza due to unidentified influenza virus with pneumonia
  - J11.00 Influenza due to unidentified influenza virus with unspecified type of pneumonia
  - J11.08 Influenza due to unidentified influenza virus with specified pneumonia
- J11.1 Influenza due to unidentified influenza virus with other respiratory manifestations
- J11.2 Influenza due to unidentified influenza virus with gastrointestinal manifestations
- J11.8 Influenza due to unidentified influenza virus with other manifestations
  - J11.81 Influenza due to unidentified influenza virus with encephalopathy
  - J11.82 Influenza due to unidentified influenza virus with myocarditis
  - J11.83 Influenza due to unidentified influenza virus with otitis media
  - J11.89 Influenza due to unidentified influenza virus with other manifestations
- G03.0 Nonpyogenic meningitis
- G03.1 Chronic meningitis
- G03.2 Benign recurrent meningitis [Mollaret]
- G03.8 Meningitis due to other specified causes
- G03.9 Meningitis, unspecified
- F07.81 Postconcussional syndrome
- G93.2 Benign intracranial hypertension
- F41.0 Panic disorder [episodic paroxysmal anxiety
- G50 (G50.0, 1, 8, 9) Disorders of trigeminal nerve
- [2022 ICD-10-CM Codes V00-Y99: External causes of morbidity (icd10data.com)](https://www.icd10data.com/ICD10CM/Codes/V00-Y99)
