# Supplementary material for: Impact of an Electronic Health Record–Based Interruptive Alert Among Patients With Headaches Seen in Primary Care: Cluster Randomized Controlled Trial
Source: JMIR Med Inform. 2024 Aug 29;12:e58456. doi: 10.2196/58456 (PMC11376138; doi:10.2196/58456)
Supplement: Multimedia Appendix 3 [file medinform-v12-e58456-s003.docx]

**Multimedia Appendix 3.** Snapshots of the electronic alert questionnaire and Express Lane or SmartSet tool.

Snapshot of the electronic alert (Best Practice Advisory) based questionnaire for collecting information about headache characteristics, along with the links to the Smart Set guide, and electronic provider-to-provider consultation to neurology.


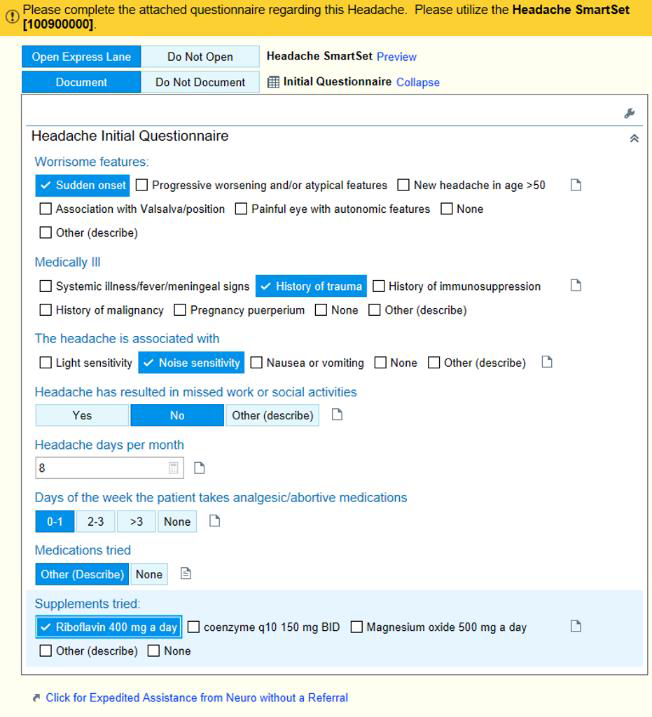


This image carriers an Epic’s copyright statement: © 2024 Epic Systems Corporation.

Express Lane/Smart Set tool for providers to guide medication management including dosage and tapering schedules, and quick options to place laboratory and imaging orders.


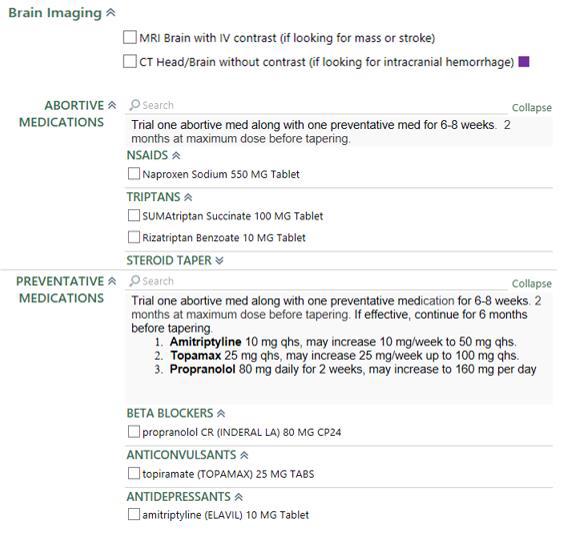


This image carriers an Epic’s copyright statement: © 2024 Epic Systems Corporation.
