# Supplementary material for: Impact of an Electronic Health Record–Based Interruptive Alert Among Patients With Headaches Seen in Primary Care: Cluster Randomized Controlled Trial
Source: JMIR Med Inform. 2024 Aug 29;12:e58456. doi: 10.2196/58456 (PMC11376138; doi:10.2196/58456)
Supplement: Multimedia Appendix 4 [file medinform-v12-e58456-s004.docx]

**Multimedia Appendix 4.** Survey administered by Geisinger’s Survey Research and Recruitment Core (SRRC).

| **Variable Name** | **Question** | **Response Options** | **Comments / Skips** |
| --- | --- | --- | --- |
| GREET | Hello my name is [Survey Administrator Name] and I am calling from Geisinger. | KEY |  |
| VERIFY | May I please speak with [Patient Name] ? | 1 = Yes 2 = No | If (ans = 1) skip to INTRO |
| NOT_THERE | I’m sorry, but I need to speak to [Patient Name] directly. Is there a better time to call? (set callback)  Thank you for your time. | Set callback | End Call. |
| INTRO | Hello [Patient Name]. Do you have a few minutes to discuss a recent appointment to [Clinic Name]? | 1 = Yes 2 = No | If 1, go to INTRO2 If 2, go to INTRO1 |
| INTRO1 | Is there a better time to call? | 1 = Yes 2 = No | If (ans = 1) set callback time If (ans = 2), enter comment from patient and skip to THANK_YOU_REFUSAL |
| INTRO2 | Our records indicate that you recently were seen by [Provider] at [Clinic Name] on [Date]. Is this correct? | 1 = Yes 2 = No, wrong provider 3 = No, wrong clinic 4 = No, wrong date 5 = I don't remember | If 1, go to INTRO3 If 2, 3, or 4, enter comment from patient and skip to THANK YOU_WRONG If 5, go to INTRO_UNSURE |
| INTRO_UNSURE | Okay, according to our records, your [HEADACHE/MIGRAINE COMPLAINT] was addressed during this visit. Does that ring any bells? | 1 = Yes 2 = No | If 1, skip to INTRO4 If 2, skipt to THANK_YOU_WRONG |
| INTRO3 | Great. Also, according to our records, your [HEADACHE/MIGRAINE COMPLAINT] was addressed during this visit. Is this correct as well? | 1 = Yes 2 = No 3 = I Don't Remember | If 1, go to INTRO4 If 2 or 3, enter comment from patient and skip to THANK YOU_WRONG |
| INTRO4 | Great. Based upon your response, we would like to ask you a few questions about your experiences with your [HEADACHE/MIGRAINE COMPLAINT]. If you agree to take part in this interview, the information collected is part of a study to understand how well a new tool we are using in the clinic is working. The information you provide will help us care for people in the future who are diagnosed with headache/Migraine.  The questions should take approximately 5-10 minutes of your time.  There are no right or wrong answers. We want to hear about your experiences. Please let me know if any question does not make sense. If there is a question you don’t want to answer please let me know and I’ll move to the next question. You can decline to answer any question that you want.   We do not share your personal or medical information outside the study team so there is little risk to you to participate. Answering this survey may help others in the future.  If you have any questions or concerns, you can reach out to Dr. Apoorva Pradhan at ---.  Would you like to proceed to the questions?   To restate, on [date] you were seen by your provider for [HEADACHE/MIGRAINE COMPLAINT]. In the Questions that follow, we will refer to your [HEADACHE/MIGRAINE COMPLAINT] as "headache". I am going to begin the questions now. | 1 = Yes 2 = No | If 1, go to Q1 If 2, enter comment from patient and skip to THANK_YOU_REFUSAL |
| Q1 | When you have headaches, how often is the pain severe? | 1) Never 2) Rarely 3) Sometimes 4) Very Often 5) Always | 1) Never = 6 points 2) Rarely = 8 points 3) Sometimes = 10 points 4) Very Often = 11 points 5) Always = 13 points |
| Q2 | How often do headaches limit your ability to do usual daily activities including household work, work, school, or social activities? | 1) Never 2) Rarely 3) Sometimes 4) Very Often 5) Always | 1) Never = 6 points 2) Rarely = 8 points 3) Sometimes = 10 points 4) Very Often = 11 points 5) Always = 13 points |
| Q3 | When you have a headache, how often do you wish you could lie down? | 1) Never 2) Rarely 3) Sometimes 4) Very Often 5) Always | 1) Never = 6 points 2) Rarely = 8 points 3) Sometimes = 10 points 4) Very Often = 11 points 5) Always = 13 points |
| Q4 | In the past 4 weeks, how often have you felt too tired to do work or daily activities because of your headaches? | 1) Never 2) Rarely 3) Sometimes 4) Very Often 5) Always | 1) Never = 6 points 2) Rarely = 8 points 3) Sometimes = 10 points 4) Very Often = 11 points 5) Always = 13 points |
| Q5 | In the past 4 weeks, how often have you felt fed up or irritated because of your headaches? | 1) Never 2) Rarely 3) Sometimes 4) Very Often 5) Always | 1) Never = 6 points 2) Rarely = 8 points 3) Sometimes = 10 points 4) Very Often = 11 points 5) Always = 13 points |
| Q6 | In the past 4 weeks, how often did headaches limit your ability to concentrate on work or daily activities? | 1) Never 2) Rarely 3) Sometimes 4) Very Often 5) Always | 1) Never = 6 points 2) Rarely = 8 points 3) Sometimes = 10 points 4) Very Often = 11 points 5) Always = 13 points |
| Q7 | On how many days in the last 3 months did you have a headache? If a headache lasted more than 1 day, count each day. | 0) 0 1) 1 2) 2 3) 3 4) 4 5) 5 6) 6 7) 7 8) 8 9) 9 10) 10 11) 11 12) 12 13) 13 14) 14 15) 15 16) 16 17) 17 18) 18 19) 19 20) 20 21) 21 22) 22 23) 23 24) 24 25) 25 26) 26 27) 27 28) 28 29) 29 30) 30 31) 31 | If (Q7 ans ≥ 12), continue to Q8  If (Q7 ans < 12), summate the points from Q1 through Q6.  If (total points for Q1 through Q6 ≥ 50), continue to Q8  If (total points for Q1 through Q6 < 50), skip to DISQUALIFY |
| Q8 | On a scale of 0 - 10, on average how painful were these headaches? (where 0=no pain at all, and 10=pain as bad as it can be.) | 0) 0 1) 1 2) 2 3) 3 4) 4 5) 5 6) 6 7) 7 8) 8 9) 9 10) 10 |  |
| Q9 | Are you currently prescribed any medications for the management of your headache? | 1) yes 2) no | If (ans = 2), skip to INTRO BRIEF AND DEMOGRAPHICS |
| Q10 | Are you able to quickly return to your normal activities (i.e. work, family, leisure, social activities) after taking your headache medication? | 1) yes 2) no |  |
| Q11 | Does one dose of your headache medication usually relieve your headache and keep it away for at least 24 hours? | 1) yes 2) no |  |
| Q12 | Are you comfortable enough with your headache medication to be able to plan your daily activities? | 1) yes 2) no |  |
| INTRO BRIEF AND DEMOGRPAHICS | Next, we would like to ask you a few questions about yourself since it's important to understand patients' characteristics when reviewing all information collected. This information will only be used for the study and only the study team members will have access to it. |  |  |
| DEM1 | What is the highest level of education you have finished? | 1) < High school/GED 2) Highschool/ GED 3) Some College or technical program 4) 4 - year college (BS/BA) 5) Master's Degree (MS/MA/MPH) 6) Doctorate (PhD/ScD or Professional (MD/DO/JD) 7) Refuse |  |
| DEM2 | What is your yearly household income? | 1) $25,000 or less 2) Over $25,000-$50,000 3) Over $50,000-$100,000 4) Over $100,000 5) Refuse |  |
| QUALIFY | Based on your responses, you qualify for 2 follow-up surveys, which would occur 3 months from now and 6 months from now. The surveys would each take 5-10 minutes.   Would you like to remain in the study and be contacted for these follow-up surveys? | 1) yes 2) no | If (ans = 1), skip to THANK_YOU_YES If (ans = 2), go to THANK_YOU_NO |
| THANK_YOU_YES | Thank you so much for participating in this survey and sharing your experiences with us. We appreciate the time you have already given us as well as your continued participation. You will be contacted by Geisinger again in 3 months for the next survey. Thank you and have a great day. |  | End call. |
| THANK_YOU_NO | We will make a note in our records not to contact you for further surveys related to this study. Thank you so much for participating in this survey and sharing your experiences with us. We appreciate the time you have given us, and we hope you have a great day. |  | End call. |
| DISQUALIFY | This completes the questions. Thank you so much for participating in this survey and sharing your experiences with us. We appreciate your time and hope you have a great day. |  | End call. |
| THANK_YOU_REFUSAL | Since you have indicated you would not like to discuss your appointment, we will make a notation in our records to not contact you further about this survey. Thank you for your time and have a great day. | KEY | End Call. |
| THANK_YOU_WRONG | I apologize for the confusion. We will look back into our records, and possibly contact you again in the future. Thank you for your time and have a great day. | KEY | End Call. |
