# Supplementary material for: Impact of an Electronic Health Record–Based Interruptive Alert Among Patients With Headaches Seen in Primary Care: Cluster Randomized Controlled Trial
Source: JMIR Med Inform. 2024 Aug 29;12:e58456. doi: 10.2196/58456 (PMC11376138; doi:10.2196/58456)

**Multimedia Appendix 5.** Distribution of pain scores and the number of headache days observed in the intervention and control arm of the study.

This figure represents the skewed distribution of the number of headache days observed in the intervention and control arm of the study.


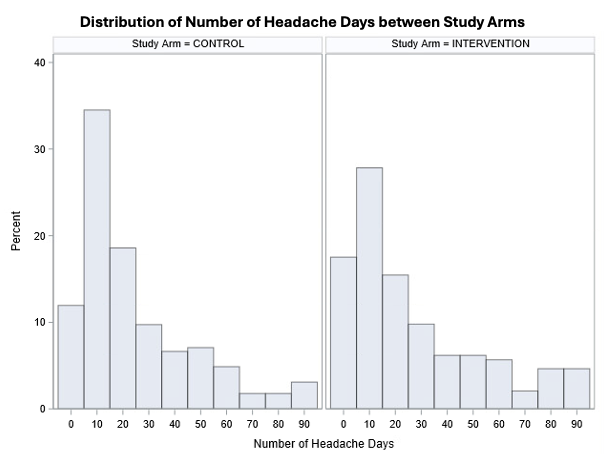


This figure represents the skewed distribution of pain scores observed in the intervention and control arm of the study.


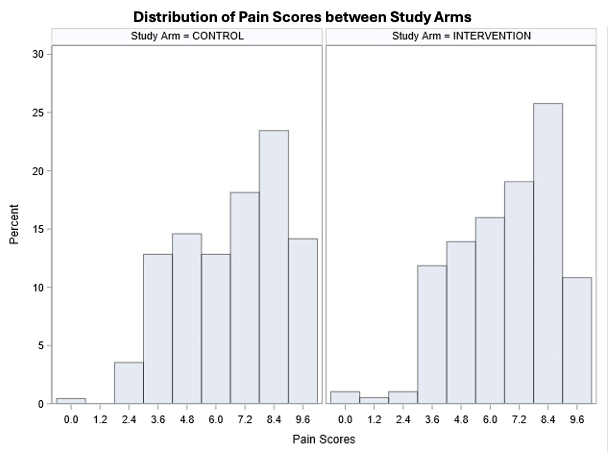

Supplement: Multimedia Appendix 5 [file medinform-v12-e58456-s005.docx]
